# Brassica yellows virus P0 protein impairs the antiviral activity of NbRAF2 in *Nicotiana benthamiana*

**DOI:** 10.1093/jxb/ery131

**Published:** 2018-04-05

**Authors:** Qian Sun, Yuan-Yuan Li, Ying Wang, Hang-Hai Zhao, Tian-Yu Zhao, Zong-Ying Zhang, Da-Wei Li, Jia-Lin Yu, Xian-Bing Wang, Yong-Liang Zhang, Cheng-Gui Han

**Affiliations:** 1 State Key Laboratory for Agro-biotechnology and Ministry of Agriculture Key Laboratory of Plant Pathology, China Agricultural University, Beijing, P. R. China; 2 State Key Laboratory of Agro-Biotechnology and Ministry of Agriculture Key Laboratory of Soil Microbiology, College of Biological Sciences, China Agricultural University, Beijing, P. R., China

**Keywords:** Brassica yellows virus, NbRAF2, *Nicotiana benthamiana*, nuclear localization, P0, stromules

## Abstract

In interactions between poleroviruses and their hosts, few cellular proteins have been identified that directly interact with the multifunctional virus P0 protein. To help explore the functions of P0, we identified a Brassica yellows virus genotype A (BrYV-A) P0^BrA^-interacting protein from *Nicotiana benthamiana*, Rubisco assembly factor 2 (NbRAF2), which localizes in the nucleus, cell periphery, chloroplasts, and stromules. We found that its C-terminal domain (amino acids 183–211) is required for self-interaction. A split ubiquitin membrane-bound yeast two-hybrid system and co-immunoprecipitation assays showed that NbRAF2 interacted with P0^BrA^, and co-localized in the nucleus and at the cell periphery. Interestingly, the nuclear pool of NbRAF2 decreased in the presence of P0^BrA^ and during BrYV-A infection, and the P0^BrA^-mediated reduction of nuclear NbRAF2 required dual localization of NbRAF2 in the chloroplasts and nucleus. *Tobacco rattle virus*-based virus-induced gene silencing of *NbRAF2* promoted BrYV-A infection in *N. benthamiana*, and the overexpression of nuclear NbRAF2 inhibited BrYV-A accumulation. *Potato leafroll virus* P0^PL^ also interacted with NbRAF2 and decreased its nuclear accumulation, indicating that NbRAF2 may be a common target of poleroviruses. These results suggest that nuclear NbRAF2 possesses antiviral activity against BrYV-A infection, and that BrYV-A P0^BrA^ interacts with NbRAF2 and alters its localization pattern to facilitate virus infection.

## Teaser

The Brassica yellows virus genotype A P0 protein interacts with Rubisco assembly factor 2 in tobacco, affecting its nuclear accumulation and promoting viral infection.

## Introduction

Numerous studies have demonstrated that chloroplasts are a common target of many plant viruses. Chloroplasts and their components are involved in viral movement, replication, and symptom development, and also participate in plant defense against viruses (Zhao *et al.*, 2016; Bhattacharyya and Chakraborty, 2018). Several chloroplast proteins have been shown to play negative roles in viral pathogenesis. The coat protein of *Alfalfa mosaic virus* interacts with the photosystem-II oxygen-evolving complex protein PsbP, and overexpression of PsbP markedly reduces virus accumulation (Balasubramaniam *et al.*, 2014). The cylindrical inclusion protein from *Plum pox virus* (PPV) binds to the photosystem-I PSI-K protein, which is the product of *psaK*. PPV infection down-regulates the expression of *psaK* mRNA in inoculated leaves, and the silencing of *psaK* leads to a greater accumulation of PPV (Jiménez *et al.*, 2006). Pathogens attempt to intercept chloroplast proteins by interacting and sequestering them in the cytosol before they are imported into the chloroplasts or by affecting their normal activities (Jin *et al.*, 2007; Shi *et al.*, 2007; Balasubramaniam *et al.*, 2014; Kong *et al.*, 2014).

In mammals, the single pterin-4a-carbinolamine dehydratase/dimerization co-factor of hepatocyte nuclear factor 1 (PCD/DCoH) acts as a metabolic enzyme, and has PCD activity in the mitochondria and also acts as a DCoH in the nucleus (Suck and Ficner, 1996). The activities of PCD and DCoH are independent (Rhee *et al.*, 1997**).** In plants, there are two copies of PCD/DCoH homologs (Naponelli *et al.*, 2008). Type 1 is localized to the mitochondria and has PCD activity, whereas type 2, without PCD activity, is directed to the chloroplast (Naponelli *et al.*, 2008). The Arabidopsis protein Rubisco assembly factor 2 (AtRAF2) (Fristedt *et al.*, 2018), also named SDIRIP1 (Zhang *et al.*, 2015*a*) or ATP1 (Oh *et al.*, 2017), is a type 2 protein of the PCD/DCoH family (Naponelli *et al.*, 2008). AtRAF2 is nuclear-encoded and localized to the chloroplast (Naponelli *et al.*, 2008; Zybailov *et al.*, 2008). It has been suggested that AtSDIRIP1/AtRAF2 is localized not only to the chloroplast but also to the cell periphery and nucleus (Zhang *et al.*, 2015*a*). The chloroplast/nuclear protein AtSDIRIP1/AtRAF2 interacts with the E3 ligase SDIR1 in the cytosol, and is subsequently degraded by it. AtSDIRIP1/AtRAF2 selectively regulates the expression of the abscisic acid (ABA)-responsive transcription factor gene *ABA-INSENSITIVE5* to regulate ABA-mediated seed germination and salt-stress responses (Zhang *et al.*, 2015*a*). The cytosolic RING-type E3 ligase AtAIRP2 also targets AtATP1/AtRAF2 for degradation, and AtAIRP2 and AtSDIR1 play a combinatory role in ABA- and salt-stress responses in Arabidopsis (Oh *et al.*, 2017). In addition, *Zea mays* RAF2 (ZmRAF2) shares a high amino acid sequence identity with AtRAF2 (Naponelli *et al.*, 2008), and both proteins are involved in Rubisco assembly (Feiz *et al.*, 2014; Fristedt *et al.*, 2018). However, in plants, the function of RAF2 in the nucleus remains unknown.

Poleroviruses, belonging to the family Luteoviridae, infect many crops of economic importance and cause serious yield losses (Taliansky *et al.*, 2003; Stevens *et al.*, 2005). They cause yellowing symptoms in a wide range of hosts (Scagliusi and Lockhart, 2000; Peter *et al.*, 2009; Xiang *et al.*, 2011; Chen *et al.*, 2016). Viruses of this family have a positive-sense RNA genome of 5000–6000 nt, from which the P0 protein encoded by ORF0 has been shown to suppress RNA silencing (Pfeffer *et al.*, 2002; Taliansky *et al.*, 2003; Stevens *et al.*, 2005; Pazhouhandeh *et al.*, 2006; Mangwende *et al.*, 2009; Csorba *et al.*, 2010; Han *et al.*, 2010; Kozlowska-Makulska *et al.*, 2010; Delfosse *et al.*, 2014; Zhuo *et al.*, 2014; Chen *et al.*, 2016). The P0 proteins can interact with S-phase kinase-associated protein 1 (SKP1), a member of the SKP1–Cullin 1–F-box E3 ubiquitin ligase complex, and trigger the ubiquitylation and degradation of Argonaute1 (AGO1) in plants (Pazhouhandeh *et al.*, 2006; Bortolamiol *et al.*, 2007; Csorba *et al.*, 2010; Fusaro *et al.*, 2012). P0 also physically interacts with AGO1 in the nucleus, where ASK1/2 and AtCUL1 localize, which supports the hypothesis that AGO1 could be a direct target of P0 (Bortolamiol *et al.*, 2007). However, AGO1 degradation by P0 is blocked by the inhibition of autophagy (Derrien *et al.*, 2012) but not proteasomes (Baumberger *et al.*, 2007). In addition, the P0 proteins induce cell death within the infiltration patch in *Nicotiana* species (Mangwende *et al.*, 2009; Csorba *et al.*, 2010; Fusaro *et al.*, 2012; Wang *et al.*, 2015). *Turnip yellows virus* P0^Tu^, *Potato leafroll virus* (PLRV) P0^PL^, and *Cucurbit aphid-borne yellows virus* P0^CA^ elicit a hypersensitive response in the *N. glutinosa* accession TW59 (Wang *et al.*, 2015). A genetic analysis showed that P0^Tu^ is recognized by a resistance gene, designated *Resistance to Poleroviruses 1*, and functions in an E3 ubiquitin ligase complex as a potential trigger of Resistance to Poleroviruses 1-mediated effector-triggered immunity (Wang *et al.*, 2015). However, few host proteins that directly interact with the P0 protein have been identified (Pazhouhandeh *et al.*, 2006; Bortolamiol *et al.*, 2007).

Brassica yellows virus (BrYV), a newly identified polerovirus, infects crucifer crops in China and causes yellowing or leaf-roll symptoms (Xiang *et al.*, 2011). BrYV has three genotypes, BrYV-A, B, and C (Xiang *et al.*, 2011; Zhang *et al.*, 2014), and the full-length infectious cDNA clones of these three genotypes have been developed successfully (Zhang *et al.*, 2015*b*). The BrYV P0 is a strong viral suppressor of RNA silencing and interacts with the SKP1 from *N. benthamiana* (Xiang and Han, 2011). In the present study, we successfully obtained a novel P0^BrA^-interacting protein, NbRAF2, which localized to the nucleus, cell periphery, chloroplasts, and stromules. We demonstrated that P0^BrA^ decreased the nuclear accumulation of NbRA2. The accumulation of BrYV-A increased when *NbRAF2* was silenced using *Tobacco rattle virus* (TRV)-based virus induced gene silencing (VIGS) but decreased when nuclear NbRAF2 was overexpressed. PLRV P0^PL^ also interacted with NbRAF2 and decreased its nuclear pool, indicating that nuclear NbRAF2 may be a common target of polerovirus P0s.

## Materials and methods

### Plant material and growth conditions

Wild-type *Nicotiana benthamiana* and transgenic lines with the ferredoxin NADP(H) oxidoreductase transit peptide fused to enhanced green fluorescent protein (FNR–EGFP) (Schattat *et al.*, 2011) were grown at 24 °C with a 16-h light/8-h dark cycle.

### Plasmid constructs

All of the primers used in this study are listed in Supplementary Table S1 at *JXB* online.

The vectors pGD and pGDG (Goodin *et al.*, 2002) were used for transient expression. The P38 protein encoded by *Turnip crinkle virus* was cloned into pGD for transient expression. P0^BrA^ was cloned into to pGD–3Flag, a modified version of vector pGD that has a C-terminal-fused 3×Flag tag. For co-immunoprecipitation (Co-IP) and confocal microscopy, pGD–3G-mCherry was constructed. A DNA fragment of GGG-mCherry was amplified and cloned into the vector pGD to produce pGD–3G-mCherry. Full-length AtRAF2, NbRAF2, and their mutants were independently cloned into the vector pGDGm [a modified version of pGD that allows the production of a C-terminal green fluorescent protein (GFP)-fused protein] and pGD–3G-mCherry. NbSKP1 was cloned into the vector pGDGm. For its subcellular localization, P0^BrA^ was amplified and cloned into pSuper1300–GFP (Yang *et al.*, 2010). Wild-type BrYV full-length cDNAs were cloned into the pCB301-2 × 35S-MCS-HDV_RZ_-NOS vector (Yao *et al.*, 2011; Zhang *et al.*, 2015*b*).

### *Agrobacterium*-mediated transient expression in *N. benthamiana*

Plasmids were transformed into the *Agrobacterium tumefaciens* strain EHA105 or C58CI using the freeze–thaw method (Holsters *et al.*, 1978). The recombinant EHA105 or C58CI was grown overnight, resuspended in infiltration buffer (10 mM MgCl_2_, 10 mM MES, and 100 μM acetosyringone), and incubated at room temperature for at least 3 h before infiltration. The *A. tumefaciens* cultures were infiltrated into *N. benthamiana* leaves and the infiltrated leaves were detached for the corresponding assays.

### Yeast two-hybrid (Y2H) screen and interaction assays

For the Y2H assay, the Clontech Matchmaker GAL4 Two-Hybrid System 3 (Clontech, Mountain View, CA, USA) was used. Protein-interacting screens were performed with the Matchmaker GAL4 two-hybrid system according to the manufacturer’s protocol. The full-length of P0^BrA^ was cloned into pGDBKT7 containing a binding domain (BD) to generate BD–P0^BrA^ and then transformed into the yeast host strain Y187. The Arabidopsis cDNA library was used to screen P0^BrA^-binding proteins. The full-length AtRAF2 was cloned into pGDADT7 containing an activating domain (AD) to generate AD–AtRAF2 and then transformed into the yeast host strain AH109. Co-transformants were plated onto synthetic dropout (SD) media lacking Trp and Leu (SD/−WL) and SD media lacking Ade, His, Trp, and Leu (SD/−AHLW). Full-length NbRAF2 (GenBank accession MG560271), AtRAF2 (GenBank accession AED96036.1), NbRbcL, P0^MA^, and P0^Sc^ were independently cloned into pGDBKT7. Full-length NbRAF2, truncated NbRAF2 mutants, AtRAF2 and AtRAF2 mutants were independently cloned into pGDADT7. Protein interactions were tested using the yeast mating assay. The interaction of NbRAF2 with P0^BrA^ was identified using the split ubiquitin membrane-bound yeast two-hybrid (MbY2H) system. NbRAF2 was cloned into the MbY2H bait vector pBT3-STE, and P0^BrA^ was cloned into the prey vector pPR3-N. All combinations were transformed to the yeast strain NMY51 for mating and the transformed yeast cells were then transferred to SD/−WL and SD/−AHLW plates for 3–5 d.

### Protein extraction and western blots

Total proteins were extracted from infiltrated lamina of *N. benthamiana* leaves using 2× sodium dodecyl sulfate (SDS) sample buffer [100 mM Tris (pH 6.8), 4% (w/v) SDS, 20% (v/v) glycerol, and 0.2% (w/v) bromophenol blue]. Proteins were separated with SDS polyacrylamide gel electrophoresis. Western blots were performed with the primary anti-Flag antibody (Sigma-Aldrich) diluted at 1:1000, anti-NbRAF2 antibody diluted at 1:500, anti-GFP antibody diluted at 1:3000, anti-H3 antibody (Sigma-Aldrich) diluted at 1:3000, anti-PEPC antibody diluted at 1:3000, or anti-BrYV-A coat protein (CP) antibody diluted at 1:500, and then incubated with anti-rabbit alkaline phosphatase (Sigma-Aldrich) or anti-rabbit goat HRP secondary antibody (1:3000; Bio-Rad, Hercules, CA, USA). Finally, the membrane was detected with an enhanced chemiluminescence detection kit (GE Healthcare, Buckinghamshire, UK) according to the manufacturer’s instructions.

### Co-IP assay

The Co-IP assays were performed as previously reported (Win *et al.*, 2011) with minor modifications. Total proteins were extracted from *N. benthamiana* leaf tissues that were ground in liquid nitrogen and homogenized in 2 ml g^−1^ extraction buffer [10% (v/v) glycerol, 25 mM Tris-HCl (pH 7.5), 1 mM EDTA, 150 mM NaCl, 2% (w/v) PVPP, 10 mM DTT, 1× protease inhibitor cocktail (Sigma-Aldrich), and 0.1% (v/v) Triton X-100 (Sigma-Aldrich)]. After centrifugation at 3000 *g* for 10 min at 4 °C and filtration with a 0.45-mm filter, the clarified lysate was incubated with 4% BSA-pre-blocked anti-Flag M2 agarose beads (Sigma-Aldrich) for 3 h, and the complex was washed three times with IP buffer [10% (v/v) glycerol, 25 mM Tris-HCl (pH 7.5), 1 mM EDTA, 150 mM NaCl, and 0.1% (v/v) Triton X-100]. The immunoprecipitates were denatured and subjected to immunoblotting using corresponding antibodies.

### RNA extraction and RT-PCR

Total RNA was extracted using TRIzol Reagent (Invitrogen) according to the manufacturer’s protocol. A 4-μg of total RNA was treated with RNase-free DNase I (TaKaRa). First-strand cDNA was synthesized using 2 μg of treated RNA, oligo d(T) primer, or gene-specific primer and M-MLV Reverse Transcriptase (Promega), as instructed by the protocol. The mRNAs of *EF1A*, *NbRAF2*, and BrYV-A RNA were determined using specific primers (see Supplementary Table S1).

### Fluorescence microscopy

Agro-infiltrated leaf tissue from *N. benthamiana* was detached 2 d post infiltration (dpi), and confocal laser scanning microscopy was performed using a Leica SP8 laser-scanning microscope. GFP, mCherry, and chloroplast fluorescence were obtained using laser excitation at 488, 552, and 638 nm, respectively. For bimolecular fluorescence complementation (BiFC) assays, NbRAF2 and AtRAF2 were respectively cloned into pSPYNE–35S and pSPYCE–35S (provided by J. Kudla, Universität Münster, Germany). Yellow fluorescent protein (YFP) fluorescence was obtained using 488-nm laser excitation. To determine its distribution, the nuclear-localized NbRAF2 was quantified from Z stacks and expressed as the total number of nuclear-localized NbRAF2 particles per total number of nuclei.

### VIGS assay

For the VIGS assay (Liu *et al.*, 2002*a*), a 360-bp fragment of *NbRAF2* and full-length *GFP* were cloned and inserted into pTRV2. Each of the TRV2-based constructs was transformed into *A. tumefaciens* strain GV3101. *Agrobacterium* harboring TRV1- or TRV2-derived vectors were mixed at a 1:1 ratio and infiltrated into *N. benthamiana* plants.

### Nucleocytoplasmic fractionation assay

Nucleocytoplasmic fractionation was performed using a CelLytic^TM^ PN Isolation/Extraction kit following the manufacturer’s instructions (Sigma-Aldrich). Total, nuclear, and cytoplasmic proteins were detected by immunoblotting with corresponding antibodies. The PEPC and H3 proteins were used as cytosolic and nuclear markers, respectively.

## Results

### P0^BrA^ interacts with NbRAF2

**Fig. 1.**
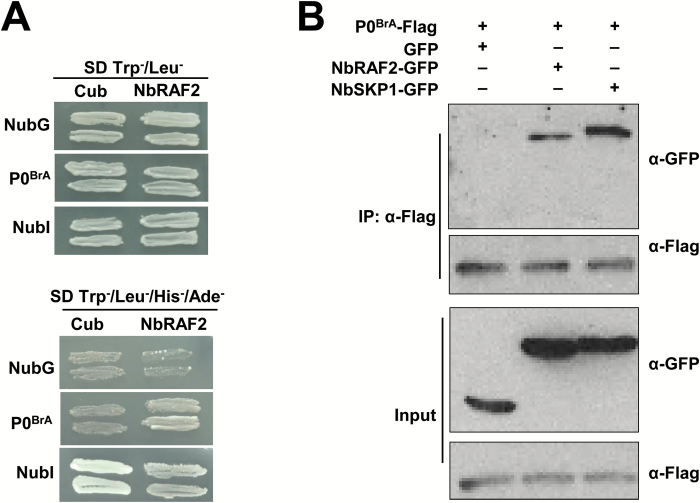
P0^BrA^ interacts with NbRAF2. (A) Analysis of interactions between NbRAF2 and P0^BrA^ using the split ubiquitin membrane-bound yeast two-hybrid (MbY2H) system. NbRAF2 was cloned into the MbY2H bait vector pBT3-STE, and P0^BrA^ was cloned into the prey vector pPR3-N. NubG constructs served as test proteins and NubI constructs were positive controls. All the combinations were transformed into yeast NMY51. Yeast strains were grown on SD/–Leu/–Trp and SD/–Ade/–His/–Leu/–Trp, and maintained at 30 °C for 2–3 d. (B) Co-immunoprecipitation analyses of NbRAF2 and P0^BrA^ proteins in *N. benthamiana* leaves. P0^BrA^–Flag was co-expressed with GFP, NbRAF2–GFP, or NbSKP1–GFP through agro-infiltration. GFP and NbSKP1 were used as negative and positive controls, respectively. Protein complexes were immunoprecipitated using anti-Flag beads. Immunoprecipitates were assessed with western blotting using anti-GFP and anti-Flag antibodies.

To study the function of the P0 protein, we performed a Y2H screen of an Arabidopsis cDNA library using P0^BrA^ as the bait to identify P0^BrA^-interacting proteins. Among the screened positive clones, one contained an intact ORF that shared 100% identity with AtRAF2 (At5g51110; GenBank accession AED96036.1). To explore the role of interactions between P0^BrA^ and RAF2 in *N. benthamiana*, we searched for AtRAF2 homologs in the *N. benthamiana* genome (http://solgenomics.net). The homolog that we identified encoded a 211-amino acid protein, shared approximately 50% identity with AtRAF2 and ZmRAF2 (GenBank accession NP_001144391) (see Supplementary Fig. S1), and was named NbRAF2 (GenBank accession MG560271). However, the traditional directed Y2H assay did not reveal a P0^BrA^–NbRAF2 interaction, although it did detect AtRAF2–P0^BrA^ (Supplementary Fig. S2). Hence, we used the split ubiquitin MbY2H system to test for an interaction (McGee, 2006; Brückner *et al.*, 2009). The P0^BrA^ was cloned into either the MbY2H bait vector to express a TF::Cub::P0^BrA^ (P0^BrA^–Cub)-fusion protein or into the MbY2H prey vector to express a NubG::P0^BrA^ (NubG–P0^BrA^)-fusion protein. NbRAF2 was also cloned into either the MbY2H bait vector to express a TF::Cub::NbRAF2 (NbRAF2–Cub)-fusion protein or into the MbY2H prey vector to express a NubG::NbRAF2 (NubG–NbRAF2)-fusion protein. A series of controls were performed. NubG constructs served as test proteins and NubI constructs were used as positive controls. All combinations were transformed into yeast cells for mating, and the transformed yeast cells were transferred to SD/−WL and SD/−AHLW plates for 2–3 d. However, the NubG–NbRAF2/Cub combination also grew on the SD/−AHLW plates (data not shown). Thus, we chose NbRAF2–Cub to identify the interaction. Yeast transformed with NbRAF2–Cub and NubG–P0^BrA^ grew on selective plates, as did the positive control; however, the negative controls did not (Fig. 1A). Thus, P0^BrA^ can directly interact with NbRAF2 in yeast.

We used Co-IP assays to further confirm whether P0^BrA^ interacted with NbRAF2 in plant cells. Flag-tagged P0^BrA^ (P0^BrA^–Flag) was co-expressed with GFP, GFP-tagged NbRAF2 (NbRAF2–GFP), or GFP-tagged NbSKP1 (NbSKP1–GFP) in *N. benthamiana* leaves through agro-infiltration. The interaction between P0^BrA^ and NbSKP1 was used as a positive control. At 2 dpi, protein complexes were immunoprecipitated using anti-Flag beads. The P0^BrA^–Flag was co-immunoprecipitated with NbRAF2–GFP, but not with GFP (Fig. 1B). These experiments demonstrated that P0^BrA^ interacts with NbRAF2 in plant cells.

### Subcellular localization of NbRAF2

**Fig. 2.**
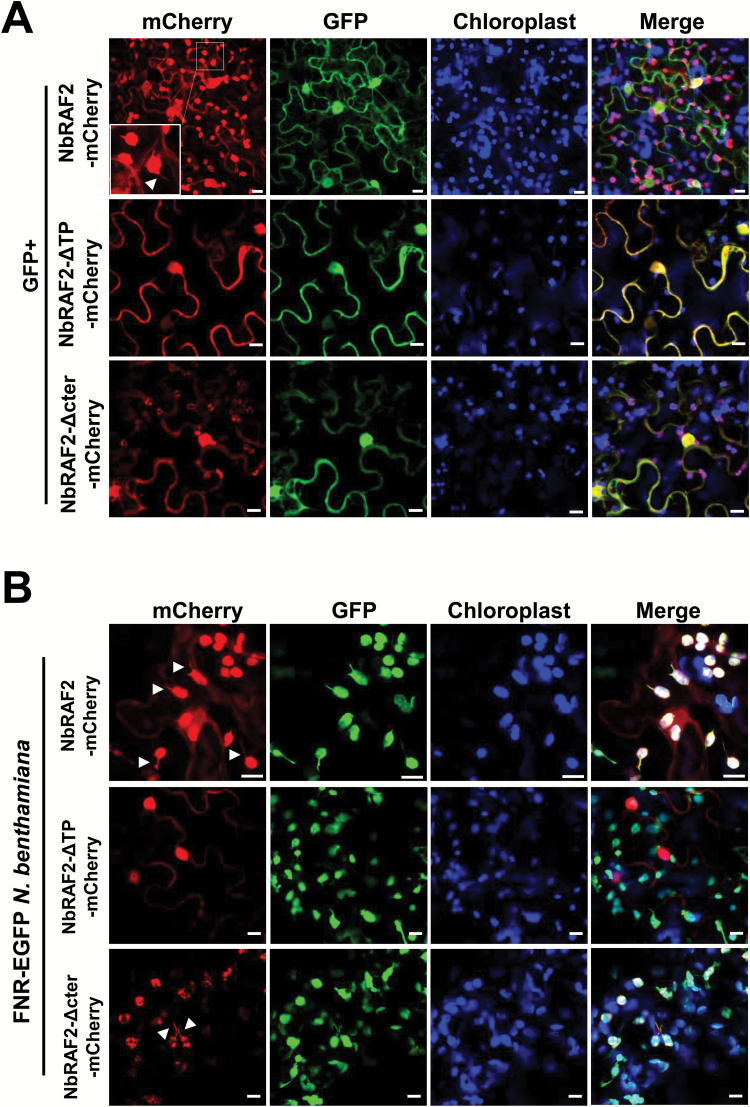
Subcellular localization of NbRAF2 and its mutants. (A) The subcellular localization of NbRAF2 and its mutants in *N*. *benthamiana* leaf cells as assessed by confocal microscopy. NbRAF2–mCherry and its mutants were transiently co-expressed with GFP in leaves through agro-infiltration and imaged at 2 d post-inoculation. The white arrowhead indicates that the protein is localized to the stromules of chloroplasts. A magnification of the selected area is shown in the first image on the top row. (B) The subcellular localization of NbRAF2 and its mutants in FNR–GFP transgenic *N. benthamiana* leaf cells as assessed with confocal microscopy. NbRAF2–mCherry and its mutants were independently transiently expressed in leaves of FNR–GFP transgenic *N. benthamiana* through agro-infiltration. White arrowheads indicate that the protein is localized to the stromules of chloroplasts. The scale bars represent 10 μm.

To investigate the subcellular localization of NbRAF2, we generated mCherry-tagged NbRAF2 (NbRAF2–mCherry) (see Supplementary Fig. S3), and then transiently co-expressed it with free GFP in *N. benthamiana* leaves through agro-infiltration. The infiltrated leaves were collected at 2 dpi and observed with confocal microscopy. The mCherry signal revealed that NbRAF2 was present in the chloroplast, cell periphery, and nucleus of the same cells, similar to the subcellular localization of AtSDIRIP1/AtRAF2. Surprisingly, NbRAF2 was localized to the chloroplast stromules (Fig. 2A). To confirm this, we transiently expressed NbRAF2–mCherry in FNR–EGFP transgenic *N. benthamiana* through agro-infiltration. The FNR–EGFP was used here as a stromule-localized marker (Schattat *et al.*, 2011). Confocal microscopy showed that NbRAF2–mCherry could perfectly co-localize with FNR–EGFP in chloroplasts and stromules (Fig. 2B). Thus, NbRAF2–mCherry was localized not only to the nucleus and cell periphery but also to the chloroplasts and stromal fraction. Similarly, AtRAF2 was also localized to the stromules (Supplementary Fig. S4).

### The C-terminal residues of NbRAF2 are required for its self-interaction

**Fig. 3.**
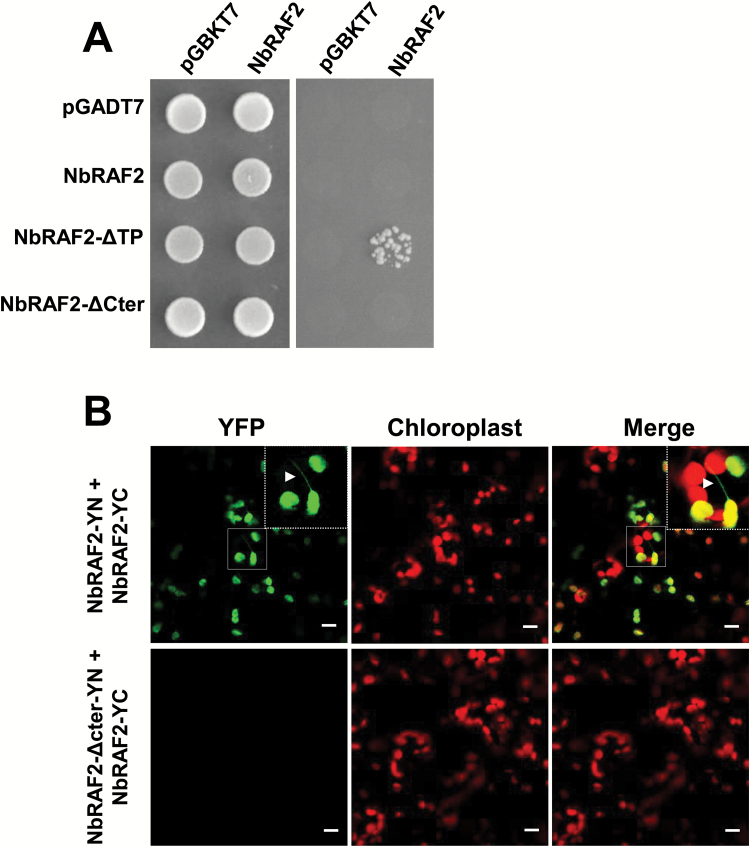
The C-terminal residues of NbRAF2 are required for its self-interaction. (A) Self-interaction analysis of NbRAF2 in yeast. Activating domain (AD) constructs containing NbRAF2 and its variants were independently transformed with BD–NbRAF2. Yeast cells transformed separately with binding domain (BD) and AD vectors showed no growth on selective plates. Yeast strains were grown on SD/–Leu/–Trp and SD/–Ade/–His/–Leu/–Trp, and maintained at 30 °C for 5–7 d. The yeast two-hybrid results showed that C-terminal residues are required for the self-interaction of NbRAF2. (B) BiFC assays for NbRAF2 self-interaction were performed on *N. benthamiana* leaves through agro-infiltration. YFP is an indicator of protein–protein interactions. NbRAF2–NbRAF2 interactions are apparent in the chloroplasts and chloroplast stromules, and the self-interaction requires C-terminal residues.

The Y2H and BiFC assays showed that NbRAF2 self-interacted. The Y2H results demonstrated that the combinations of pGBKT7–NbRAF2 and pGADT7–NbRAF2 could not grow on the SD/−AHLW selective plates (Fig. 3A), but NbRAF2 could interact with NbRAF2–ΔTP, a truncated mutant of NbRAF2 that contained a deleted transit peptide (TP) of 1–45 aa in the N-terminus (Figs 2, 3A, Supplementary Fig. S3). For the BiFC assays, NbRAF2 was fused to N- and C-terminal halves of YFP at the N-termini to generate NbRAF2–YN and NbRAF2–YC, respectively. The reconstituted YFP fluorescence was observed in *N. benthamiana* leaf epidermal cells co-infiltrated with NbRAF2–YN and NbRAF2–YC, which co-localized with chloroplast autofluorescence (Fig. 3B). In addition, YFP signals occurred in the stromules. Fluorescence could not be visualized in the negative controls NbRAF2-YN/YC and YN/NbRAF2-YC (Supplementary Fig. S5). Arabidopsis and maize RAF2 can form dimers (Valkai, 2004; Feiz *et al.*, 2014), consistent with our observations for NbRAF2. In addition, the C-terminal truncated mutant NbRAF2–ΔCter, which contained a deleted C-terminal 183–211 aa and localized to the nucleus and chloroplast (Supplementary Fig. S3, Fig. 2), failed to interact with full-length NbRAF2 (Fig. 3A, B).

Thus, NbRAF2 self-interacted in chloroplasts and stromules, and the C-terminal residues were required for self-interaction but not for chloroplastic and nuclear localization. Similarly, the C-terminal 192–220 aa of AtRAF2 was required for its self-interaction but not for its chloroplastic and nuclear localization (Supplementary Figs S4, S6).

### NbRAF2 co-localizes with P0^BrA^ in the nucleus and cell periphery

**Fig. 4.**
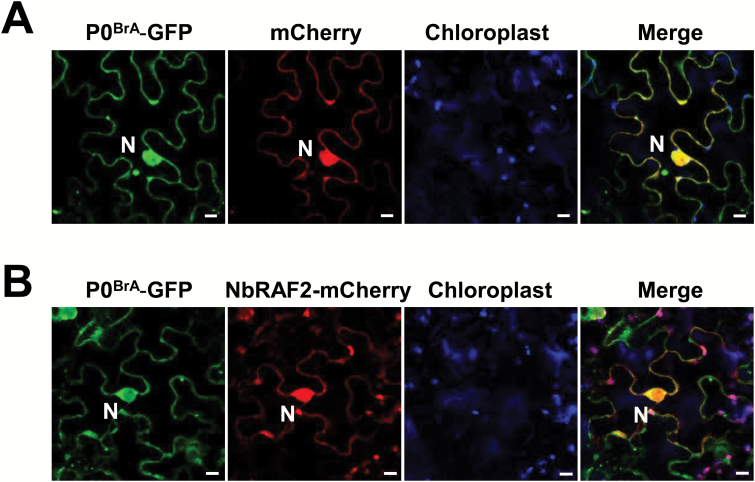
NbRAF2 co-localizes with P0^BrA^ in the nucleus and cell periphery. (A) P0^BrA^ localizes to the nucleus and the cytoplasm. GFP-tagged P0^BrA^ was transiently co-expressed with mCherry in *N. benthamiana* leaves. (B) Co-localization of NbRAF2 and P0^BrA^. The co-localization of the proteins is shown in the merged image. Confocal images show that NbRAF2 co-localizes with P0^BrA^ in the nucleus and cell periphery. Images were taken at 2 d post-inoculation. The scale bars represent 10 μm.

To investigate the subcellular localization of P0^BrA^, we constructed a vector expressing the P0^BrA^ protein with a GFP-tag fused to its C-terminus (P0^BrA^–GFP). P0^BrA^–GFP was co-expressed with free mCherry (which was used as a marker to delineate the nucleus and cytoplasm) in *N. benthamiana* leaves through agro-infiltration. Confocal microscopy showed that P0^BrA^–GFP co-localized with mCherry, suggesting that P0^BrA^–GFP can localize to the nucleus and the cell periphery (Fig. 4A). Moreover, P0^BrA^–GFP also formed punctate structures in the cytosol (Fig. 4A). To further investigate where P0^BrA^ and NbRAF2 co-localized *in vivo*, the P0^BrA^–GFP was transiently co-expressed with NbRAF2–mCherry in *N. benthamiana* leaves through agro-infiltration. Confocal microscopy showed that they co-localized in the nucleus and cell periphery (Fig. 4B).

### The nuclear pool of NbRAF2 decreases in the presence of P0^BrA^ or BrYV-A

**Fig. 5.**
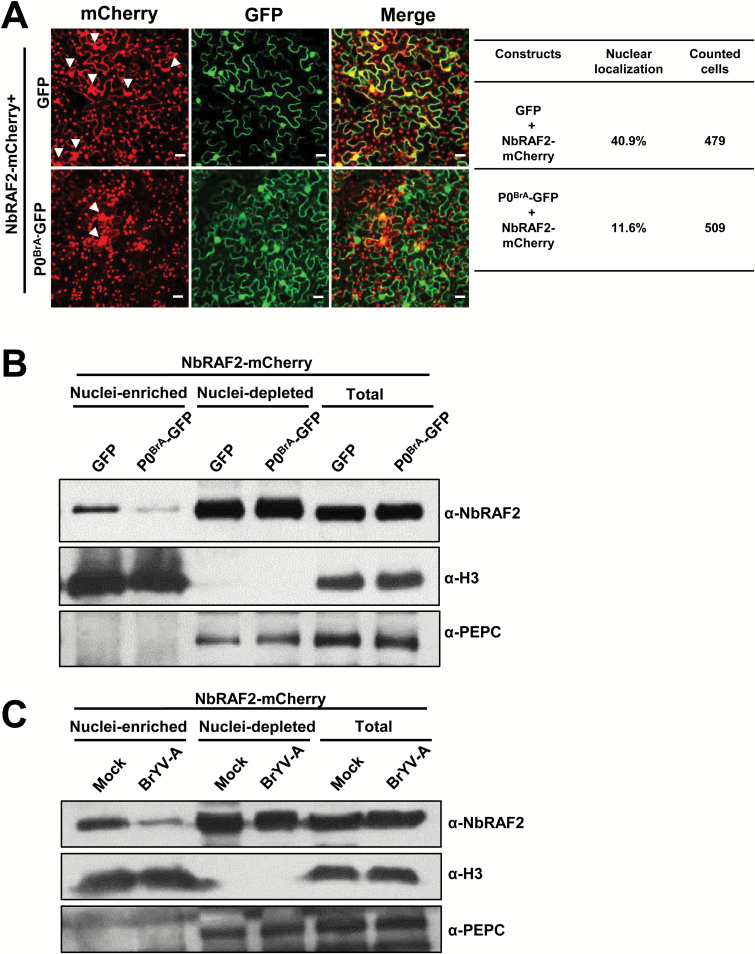
The nuclear pool of NbRAF2 decreases in the presence of P0^BrA^ or BrYV-A. (A) The localization of NbRAF2 when co-expressed with GFP or P0^BrA^–GFP. Images were taken at 2 d post-inoculation (dpi). The scale bars represent 20 μm. Z stacks of cells were imaged. Values for relative nuclear NbRAF2 are presented in the table, and are calculated as the ratio of the number of nuclear-localized NbRAF2 to the total number of nuclei, for each combination as determined by confocal microscopy. (B, C) Western blotting of NbRAF2 in nuclei-enriched and nuclei-depleted fractions, and total protein when co-expressed with GFP or P0^BrA^–GFP (B), and when co-expressed with Mock or BrYV-A (C) in *N. benthamiana* leaves. Protein samples were prepared from plant leaves at 2 dpi. All fractions were subjected to immunoblot analyses and were assessed using the corresponding antibodies. PEPC and histone H3 were used as cytosolic and nuclear markers, respectively.

Although P0^BrA^–GFP co-localized with NbRAF2–mCherry in the nucleus, the cells demonstrating nuclear co-localization were difficult to find. Interestingly, the distribution of NbRAF2–mCherry to the nucleus was significantly reduced in the presence of P0^BrA^–GFP compared with the GFP control (Fig. 5A). The relative numbers of nuclear-localized NbRAF2 were calculated based on the confocal images, and there was a reduction of approximately 30% in nuclear-localized NbRAF2 in the P0^BrA^–GFP co-expressed cells compared with GFP co-expressed cells. Nucleocytoplasmic fractionation assays were performed to further identify the nuclear accumulation of the NbRAF2 protein. The NbRAF2–mCherry with P0^BrA^–GFP or GFP were transiently co-expressed in *N. benthamiana* leaves, and protein samples were prepared at 2 dpi. A western blot analysis revealed that the nuclear pool of NbRAF2 protein significantly decreased after leaves were inoculated with P0^BrA^–GFP (Fig. 5B). However, no significant difference was observed in the total amounts of NbRAF2 protein.

NbRAF2–ΔTP, the TP deletion mutant, localized to the nucleus but not to the chloroplast; however, NbRAF2–ΔCter, the C-terminal residue deletion mutant, localized to the nucleus and the chloroplast (Fig. 2). We tested the effect of P0^BrA^ on the nuclear accumulation of these two mutants. Confocal microscopy showed that P0^BrA^ inhibited the accumulation of a nuclear pool of NbRAF2–ΔCter–mCherry, but not that of NbRAF2–ΔTP–mCherry (see Supplementary Fig. S7). Thus, the P0^BrA^-mediated reduction of nuclear NbRAF2 required the dual localization of NbRAF2 in the chloroplast and nucleus.

The effect of BrYV-A on the nuclear accumulation of NbRAF2 was assayed using nucleocytoplasmic fractionation assays and confocal microscopy. NbRAF2–mCherry was co-expressed with the mock or BrYV-A through agro-infiltration in *N. benthamiana* leaves. The samples were prepared from inoculated leaves at 2 dpi. The nuclear pool of NbRAF2 decreased after BrYV-A infection (Fig. 5C, Supplementary Fig. S8).

### *NbRAF2* expression is down-regulated during BrYV-A infection

**Fig. 6.**
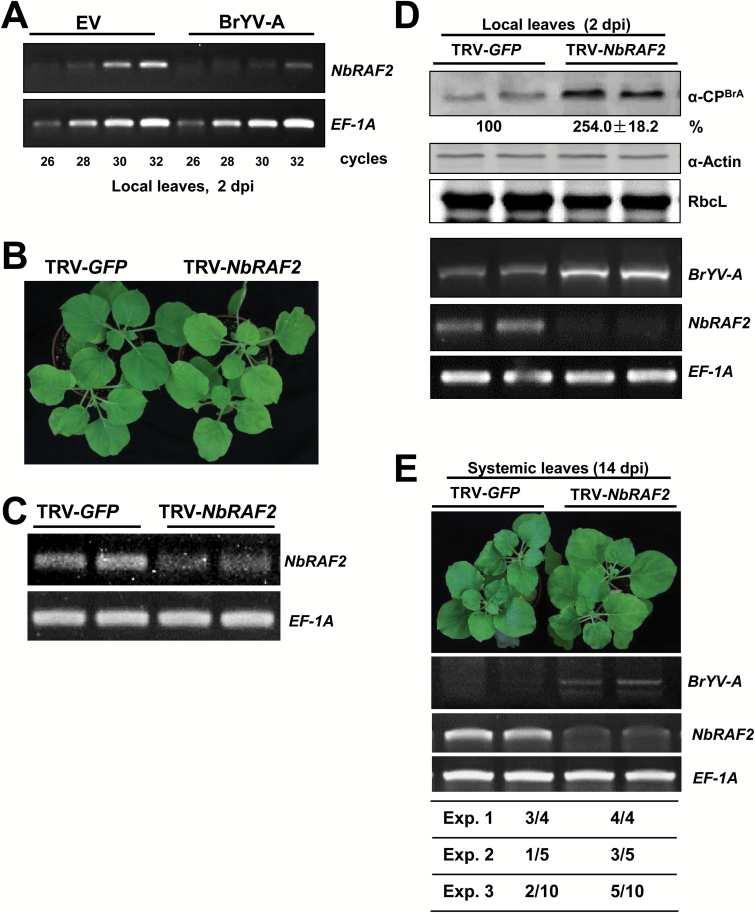
Silencing *NbRAF2* increases local BrYV-A accumulation levels in inoculated leaves and enhances systemic infection of the virus. (A) Analysis of *NbRAF2* gene expression in leaves of mock-inoculated (empty vector, EV) and BrYV-infected plants at 2 d post-inoculation (dpi). The relative accumulation of *NbRAF2* mRNA was determined using semi-quantitative RT-PCR. Images are agarose gels loaded with 26-, 28-, 30-, and 32-cycle PCR products. (B) Silencing of NbRAF2 causes leaf chlorosis compared with TRV–*GFP*-infected control plants. Images were taken ~2–3 weeks after infiltration for VIGS. (C) *NbRAF2* mRNA levels were confirmed by RT-PCR for TRV–*NbRAF2* and TRV–*GFP* systemic leaves. (D) Silencing *NbRAF2* increases local BrYV-A accumulation in inoculated leaves at 2 dpi. Proteins and RNA were extracted and subjected to western blotting and RT-PCR. The coat protein (CP) was detected with BrYV-A CP polyclonal antiserum. RbcL is the Rubisco large subunit. The actin protein was used as a loading control. ImageJ software was used to quantify the bands. RT-PCR confirmed that suppression of *NbRAF2* enhanced BrYV-A RNA levels. Representative gels of RT-PCR products are shown. (E) Silencing *NbRAF2* promotes systemic infection by BrYV-A. Photographs were taken at 14 d after BrYV-A inoculation. RT-PCR showed that *NbRAF2*-silenced plants became systemically infected with BrYV-A at 14 dpi, but non-silenced plants did not. For each RT-PCR, *EF-1A* mRNA levels were used as internal controls. The ratios below the images show the number of systemic infection plants out of the total number of infiltrated plants. The systemic infection efficiency was scored in three independent experiments.

We investigated whether the *NbRAF2* expression level was affected during BrYV-A infection. Total RNA was obtained from BrYV-A-inoculated *N. benthamiana* leaves at 2 dpi. Semi-quantitative RT-PCR showed that the mRNA level of *NbRAF2* decreased during the BrYV-A infection (Fig. 6A).

### Silencing *NbRAF2* increases the local accumulation level of BrYV-A in inoculated leaves and enhances systemic infection of the virus

To investigate the possible role of NbRAF2 during BrYV-A infection, we used the TRV-VIGS vector (Liu *et al.*, 2002*b*) to knock down the expression of *NbRAF2*. A partial cDNA fragment of NbRAF2 was cloned into the RNA2-derived vector of TRV to generate pTRV2–*NbRAF2*, and *GFP* was cloned into pTRV2 to generate pTRV2–*GFP*, which was used as a negative control. At 2–3 weeks post-inoculation, *NbRAF2*-silenced plants showed leaf chlorosis compared with TRV–*GFP*-infected control plants (Fig. 6B).The *NbRAF2* expression level was then determined with RT-PCR. The *NbRAF2* mRNA level was significantly reduced in *NbRAF2*-silenced plants compared with non-silenced controls (Fig. 6C). The RbcL protein level correspondingly decreased in *NbRAF2*-silenced plants (Fig. 6D) and RbcL interacted with NbRAF2 (see Supplementary Fig. S9), consistent with the role of ZmRAF2 and AtRAF2 in Rubisco accumulation (Feiz *et al.*, 2014; Fristedt *et al.*, 2018). Subsequently, the *NbRAF2*-silenced and non-silenced *N. benthamiana* plants were inoculated with BrYV-A using agro-infiltration. Western blot and RT-PCR analyses showed that BrYV-A CP protein and BrYV-A RNA levels increased in the *NbRAF*2-silenced plants compared with non-silenced control plants at 2 dpi (Fig. 6D). BrYV-A can cause necrosis in inoculated leaves, but symptoms are not visible in systemic leaves. However, we observed no obvious difference in necrosis of *NbRAF2*-silenced and non-silenced plants in the inoculated leaves at 3 and 4 dpi (Supplementary Fig. S10). At 14 dpi, BrYV-A was able to spread to the upper non-inoculated leaves of the *NbRAF2*-silenced plants but not easily into those of non-silenced plants. BrYV-A RNA was detected in the systemic leaves of *NbRAF2*-silenced and non-silenced plants (Fig. 6E). RT-PCR detection showed that 31.6% of non-silenced control plants had systemic BrYV-A infection at 14 dpi, but 63.2% of *NbRAF2*-silenced plants became systemically infected with BrYV-A (Fig. 6E, lower panel). These findings suggested that silencing of *NbRAF2* increases the local accumulation of BrYV-A in inoculated leaves and promotes the systemic infection of the virus. Thus, our results showed that NbRAF2 negatively regulates the BrYV-A infection.

### Overexpression of nuclear RAF2 enhances resistance to BrYV-A

**Fig. 7.**
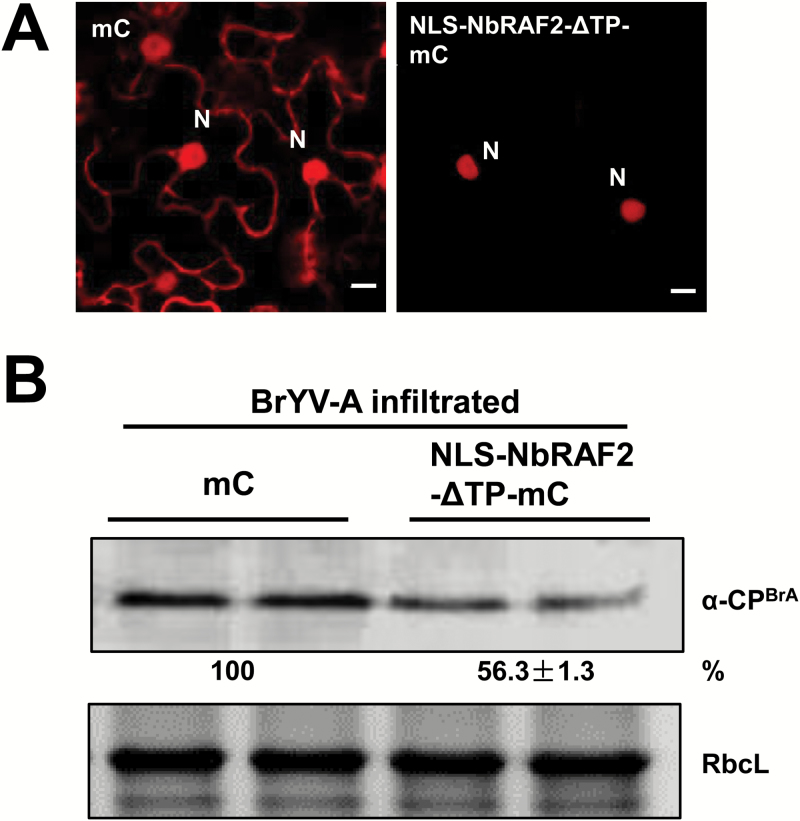
Overexpression of nuclear RAF2 enhances resistance to BrYV-A. (A) Confocal microscopy images showing the subcellular localization of mCherry (mC) and NLS–NbRAF2–ΔTP–mCherry (NLS–NbRAF2–ΔTP–mC). Images were taken at 2 d post-inoculation. The scale bars represent 10 μm. N, nucleus. (B) BrYV-A was agro-infiltrated at 1 dpi in leaves separately overexpressing NLS–NbRAF2–ΔTP–mC and mC. At 3 dpi, protein was extracted and subjected to western blotting. Coat protein (CP) was detected with BrYV-A CP polyclonal antiserum. RbcL is the Rubisco large subunit. ImageJ software was used to quantify the bands.

Silencing *NbRAF2* increased BrYV-A accumulation, and the nuclear accumulation of NbRAF2 decreased during BrYV-A infection (Figs 5C, 6D). Therefore, we investigated the function of nuclear NbRAF2 in BrYV. A nuclear localization sequence (NLS; Wen *et al.*, 1995) was fused to the N-terminus of NbRAF2–ΔTP–mCherry to generate NLS–NbRAF2–ΔTP–mCherry (see Supplementary Fig. S3). Confocal microscopy showed that NLS–NbRAF2–ΔTP–mCherry was exclusively redirected to the nucleus (Fig. 7A). Hence, we transiently expressed NLS–NbRAF2–ΔTP–mCherry and mCherry through agro-infiltration in *N. benthamiana* leaves. Then, at 1 dpi, we inoculated BrYV-A through agro-infiltration in the same leaves for 2 d. The accumulation of BrYV-A CP was examined with western blotting, which demonstrated that overexpressing nuclear NbRAF2 decreased the level of BrYV-A CP compared with mCherry (Fig. 7B). To determine whether nuclear AtRAF2 had the same role in resistance to BrYV-A, we constructed AtRAF2–ΔTP–mCherry, which was exclusively redirected to the nucleus (Supplementary Fig. S11). The results of western blotting demonstrated that overexpressing nuclear AtRAF2 also inhibited virus accumulation (Supplementary Fig. S11). These results suggested that the increased nuclear accumulation of NbRAF2 or AtRAF2 enhances resistance to BrYV-A.

## Discussion

We screened a polerovirus P0-interacting chloroplast/nucleus protein, NbRAF2, and found that it was localized not only to the cell periphery and nucleus but also to the chloroplasts and stromules. In addition, BiFC and Y2H assays showed that NbRAF2 could self-interact in chloroplasts and stromules, and the self-interaction required the 183–211 aa of its C-terminal. We obtained similar results for AtRAF2. The C-terminus of the RAF2 protein is highly conserved in *N. benthamiana*, *A. thaliana*, and *Z. mays*, indicating that the C-terminus and self-interaction may play important roles in the functions of plant RAF2 proteins. NbRAF2 interacted with NbRbcL, and *NbRAF2*-silenced plants showed reduced accumulation of NbRbcL, demonstrating that NbRAF2 may play a role similar to ZmRAF2 and AtRAF2 in Rubisco assembly (Feiz *et al.*, 2014; Fristedt *et al.*, 2018).

Increasing evidence indicates that several chloroplast proteins are localized in both chloroplasts and nuclei. These proteins have pivotal roles in plastid-to-nucleus communication (retrograde signaling), which can regulate specific changes in nuclear gene expression, including photosynthesis-related and stress-responsive genes (Koussevitzky *et al.*, 2007; Pogson *et al.*, 2008; Krause *et al.*, 2012). In response to retrograde signals, PTm, a chloroplast envelope-bound plant homeodomain PHD transcription factor, is cleaved. Its N-terminus is transported through the cytosol to the nucleus, and there it activates *ABI4* transcription in a PHD-dependent manner to regulate *Lhcb* expression (Sun *et al.*, 2011). Several proteins with dual localization in the plastids and the nucleus are involved in pathogen defense reactions (Desveaux *et al.*, 2000, 2005; Caplan *et al.*, 2008; Lai *et al.*, 2011). For instance, after NRIP1 is released from the chloroplast to the cytoplasm and the nucleus during *Tobacco mosaic virus* infection, it is then recognized by the N immune receptor to activate defenses, and this is accompanied by the induction of the hypersensitive response (Caplan *et al.*, 2008). The Why1 protein is released from the chloroplast and transported to the nucleus where it acts as a nuclear transcription factor to mediate the elicitor-induced expression of the *PR-10a* gene (Desveaux *et al.*, 2000; Isemer *et al.*, 2012).

In our study, NbRAF2 showed chloroplast/nucleus dual localization, but the dual localization mechanism and the function of NbRAF2 in the nucleus remain unknown. Interestingly, we observed that NbRAF2 was also localized to the stromules. The stromules, from which chloroplasts send out dynamic tubular extensions, aid in the transport of immune signals into the nucleus and other subcellular compartments during innate immunity (Caplan *et al.*, 2015; Gu and Dong, 2015). Stromules can be induced during immune responses and are in close contact with nuclei (Kwok and Hanson, 2004; Holzinger *et al.*, 2007; Caplan *et al.*, 2008, 2015). We hypothesized that chloroplast/nucleus NbRAF2 may be transported from the chloroplasts to the nucleus through the stromules and may have a role in transcriptional programming in the nucleus, similar to other chloroplast/nuclear proteins.

We observed that P0^BrA^ interacted and co-localized with NbRAF2 both in the nucleus and the cell periphery. The molecular mass of NbRAF2–mCherry corresponded to the predicted size of the mature NbRAF2–mCherry that was processed by the cleavage of the N-terminal chloroplast transit peptide (TP), and the nuclear isoform was the same size as the mature NbRAF2 protein, indicating that the nuclear NbRAF2 was released from chloroplasts and could interact with P0^BrA^. In addition, the subcellular distribution of NbRAF2 was altered by P0^BrA^. Confocal microscopy and the nucleocytoplasmic fractionation assay showed that P0^BrA^–GFP inhibited the accumulation of nuclear NbRAF2. AtRAF2 is degraded by the E3 ligases AtSDIR1 and AIRP2 in the cytosol when responding to ABA and high-salt stress (Zhang *et al.*, 2015*a*; Oh *et al.*, 2017). However, the total NbRAF2 level was not affected when leaves were inoculated with P0^BrA^, indicating that the reduction in the nuclear enrichment of NbRAF2 was not caused by the degradation triggered by the F-box protein P0^BrA^. Interestingly, P0^BrA^ decreased the nuclear pool of NbRAF2–ΔCter, which localized to chloroplasts and nuclei, rather than the NbRAF2–ΔTP, which localized to nuclei but not chloroplasts. This indicated that the P0^BrA^-mediated reduction of nuclear NbRAF2 requires the dual localization of NbRAF2 in chloroplasts and nuclei. We hypothesize that the chloroplast-to-nucleus translocation of NbRAF2 was regulated by chloroplast retrograde signals. Thus, P0^BrA^ interfered with the chloroplast retrograde signals to inhibit the chloroplast-to-nucleus translocation of NbRAF2. As a result, P0^BrA^ inhibited the accumulation of nuclear NbRAF2. It is unlikely that P0^BrA^ induced NbRAF2 export from the nucleus to the cytoplasm and/or chloroplast through interactions with NbRAF2 in the nucleus and cell periphery, because we observed no change in the nuclear pool of NbRAF2–ΔTP when leaves were inoculated with P0^BrA^. However, the mechanism remains elusive and will be further investigated in the future.

The nucleus of a eukaryotic cell is essential for controlling immune responses. Immune signals are transmitted to the nucleus and reprogram gene expression, which shifts the cells into defense mode (Maleck *et al.*, 2000; Tao, 2003; Dodds and Rathjen, 2010). Upon pathogen infection, the nuclear pool of immune receptors increases or immune receptors are targeted to the nucleus where they activate defense responses (Deslandes *et al.*, 2002, 2003; Shen *et al.*, 2007; Bernoux *et al.*, 2008). Other essential signaling components in the nucleus also play important roles in defense responses. The nuclear pool of the nucleo-cytoplasmic immune regulator EDS1 increases after a pathogen challenge and is essential for resistance to biotrophic and hemi-biotrophic pathogens (García *et al.*, 2010). Under unchallenged conditions, NPR1 is present in the cytosol as a stable oligomer (Mou *et al.*, 2003); however, upon pathogen infection, monomeric forms of NPR1 are transferred to the nucleus to bind the TGA family of transcription factors, activating the expression of defense-related genes, such as *PR1* and *WRKY* (Zhang *et al.*, 1999; Mou *et al.*, 2003; Wang *et al.*, 2006). We found that the nuclear pool of NbRAF2 decreased during BrYV-A infection and could negatively regulate BrYV-A infection. Silencing *NbRAF2* increased BrYV-A accumulation in the inoculated leaves and enhanced viral systemic infection. However, we cannot rule out the possibility that NbRAF2 could also be involved in BrYV-A movement. In contrast, overexpression of nuclear NbRAF2 inhibited BrYV-A accumulation in the inoculated leaves, indicating that nuclear NbRAF2 possesses antiviral activity against BrYV-A infection. Interestingly, overexpressing nuclear NbRAF2 enhanced the necrosis when inoculated with BrYV-A (Supplementary Fig. S12), which is consistent with our hypothesis that the increased resistance to BrYV-A is probably caused by the enhanced defense signaling when overexpressing RAF2 in the nucleus. Future work will address these issues. In mammals, PCD/DCoH, a homolog of plant RAF2, acts as a transcriptional co-factor to bind to, and enhance the activity of, the HNF1 transcription factor in the nucleus (Suck and Ficner, 1996). There is a good possibility that NbRAF2 acts as a transcriptional co-factor in the nucleus to regulate defense response-related genes. Thus, P0^BrA^ may affect antiviral activity of NbRAF2 through two pathways. One is that the nuclear P0 protein directly interacts with nuclear NbRAF2 to interfere with its antiviral function; another is that P0^BrA^ inhibits the accumulation of the nuclear pool of NbRAF2 by sequestering it outside the nucleus in order to enhance the viral infection. Moreover, P0 proteins encoded by PLRV, *Melon aphid-borne yellows virus*, and *Sugarcane yellow leaf virus* interacted with AtRAF2 or NbRAF2 (Supplementary Fig. S13), P0^PL^ decreased the nuclear pool of NbRAF2 (Supplementary Fig. S14), and overexpression of nuclear AtRAF2 also inhibited BrYV-A accumulation. Thus, the host RAF2 protein might be a common target of polerovirus P0 proteins.

In light of our data, we hypothesize that, under normal conditions, NbRAF2 forms dimers and participates in Rubisco assembly in the chloroplast. Nuclear NbRAF2 functions as a transcriptional co-factor to regulate defense response-related genes. Upon pathogen infection, the inactive nuclear NbRAF2 is activated to initiate expression of defensive genes. Meanwhile, chloroplasts sense the changes and transport chloroplastic immune signals, including NbRAF2, to the nucleus through stromules or other pathways to enhance resistance to pathogens. However, the effector P0 protein interacts with NbRAF2, thereby interfering with the antiviral function of nuclear NbRAF2. Subsequently, P0^BrA^ inhibits NbRAF2 nuclear accumulation by interfering with the chloroplast retrograde signals to amplify the suppression process for the the benefit of the virus, which further facilitates pathogen infection.

## Supplementary data

Supplementary data are available at *JXB* online.

Table S1. List of primers used in this research.

Fig. S1. Multiple sequence alignment of representative RAF2 proteins.

Fig. S2. Yeast two-hybrid and co-immunoprecipitation analyses demonstrating that AtRAF2 interacts with P0^BrA^.

Fig. S3. NbRAF2, AtRAF2, and their mutant constructs used for confocal microscopy or Y2H assays.

Fig. S4. The subcellular localization of AtRAF2 and AtRAF2–ΔCter.

Fig. S5. Controls for BiFC assays for NbRAF2 self-interaction.

Fig. S6. Self-interaction of AtRAF2 as demonstrated by Y2H and BiFC assays.

Fig. S7. P0^BrA^–GFP decreases the nuclear pool of NbRAF2–ΔCter but not NbRAF2–ΔTP.

Fig. S8. The localization of NbRAF2–mCherry when co-expressed with BrYV-A.

Fig. S9. Yeast two-hybrid and co-immunoprecipitation analyses demonstrating that NbRAF2 interacts with NbRbcL.

Fig. S10. Images of leaves of wild-type, *NbRAF2*-silenced, and non-silenced *N. benthamiana* inoculated with BrYV-A.

Fig. S11. Overexpression of nuclear AtRAF2 enhances resistance to BrYV-A, as demonstrated by AtRAF2–ΔTP–mCherry and western blotting.

Fig. S12. Images of leaves demonstrating that overexpression of nuclear NbRAF2 enhances necrosis when inoculated with BrYV-A.

Fig. S13. Yeast two-hybrid and co-immunoprecipitation analyses demonstrating that polerovirus P0 proteins interact with AtRAF2 and NbRAF2.

Fig. S14. P0^PL^ decreases the nuclear enrichment of NbRAF2, as demonstrated by western blotting.

Supplementary Table S1

Supplementary Figures S1-S14

## References

[CIT0001] Balasubramaniam M, Kim BS, Hutchens-Williams HM, Loesch-Fries LS 2014 The photosystem II oxygen-evolving complex protein PsbP interacts with the coat protein of *Alfalfa mosaic virus* and inhibits virus replication. Molecular Plant-Microbe Interactions 27, 1107–1118. 24940990 10.1094/MPMI-02-14-0035-R

[CIT0002] Baumberger N, Tsai CH, Lie M, Havecker E, Baulcombe DC 2007 The Polerovirus silencing suppressor P0 targets ARGONAUTE proteins for degradation. Current Biology 17, 1609–1614. 17869110 10.1016/j.cub.2007.08.039

[CIT0003] Bernoux M, Timmers T, Jauneau A, Brière C, de Wit PJ, Marco Y, Deslandes L 2008 RD19, an *Arabidopsis* cysteine protease required for RRS1-R-mediated resistance, is relocalized to the nucleus by the *Ralstonia solanacearum* PopP2 effector. The Plant Cell 20, 2252–2264. 18708476 10.1105/tpc.108.058685 PMC2553607

[CIT0004] Bhattacharyya D, Chakraborty S 2018 Chloroplast: the Trojan horse in plant–virus interaction. Molecular Plant Pathology 19, 504–518. 28056496 10.1111/mpp.12533 PMC6638057

[CIT0005] Bortolamiol D, Pazhouhandeh M, Marrocco K, Genschik P, Ziegler-Graff V 2007 The Polerovirus F box protein P0 targets ARGONAUTE1 to suppress RNA silencing. Current Biology 17, 1615–1621. 17869109 10.1016/j.cub.2007.07.061

[CIT0006] Brückner A, Polge C, Lentze N, Auerbach D, Schlattner U 2009 Yeast two-hybrid, a powerful tool for systems biology. International Journal of Molecular Sciences 10, 2763–2788. 19582228 10.3390/ijms10062763 PMC2705515

[CIT0007] Caplan JL, Kumar AS, Park E, Padmanabhan MS, Hoban K, Modla S, Czymmek K, Dinesh-Kumar SP 2015 Chloroplast stromules function during innate immunity. Developmental Cell 34, 45–57. 26120031 10.1016/j.devcel.2015.05.011 PMC4596411

[CIT0008] Caplan JL, Mamillapalli P, Burch-Smith TM, Czymmek K, Dinesh-Kumar SP 2008 Chloroplastic protein NRIP1 mediates innate immune receptor recognition of a viral effector. Cell 132, 449–462. 18267075 10.1016/j.cell.2007.12.031 PMC2267721

[CIT0009] Chen S, Jiang GZ, Wu JX, Liu Y, Qian YJ, Zhou XP 2016 Characterization of a novel Polerovirus infecting maize in China. Viruses 8, 120. 10.3390/v8050120 PMC4885075 27136578

[CIT0010] Csorba T, Lózsa R, Hutvágner G, Burgyán J 2010 Polerovirus protein P0 prevents the assembly of small RNA-containing RISC complexes and leads to degradation of ARGONAUTE1. The Plant Journal 62, 463–472. 20128884 10.1111/j.1365-313X.2010.04163.x

[CIT0011] Delfosse VC, Agrofoglio YC, Casse MF, Kresic IB, Hopp HE, Ziegler-Graff V, Distéfano AJ 2014 The P0 protein encoded by cotton leafroll dwarf virus (CLRDV) inhibits local but not systemic RNA silencing. Virus Research 180, 70–75. 24370867 10.1016/j.virusres.2013.12.018

[CIT0012] Derrien B, Baumberger N, Schepetilnikov M, Viotti C, De Cillia J, Ziegler-Graff V, Isono E, Schumacher K, Genschik P 2012 Degradation of the antiviral component ARGONAUTE1 by the autophagy pathway. Proceedings of the National Academy of Sciences, USA 109, 15942–15946. 10.1073/pnas.1209487109 PMC3465452 23019378

[CIT0013] Deslandes L, Olivier J, Peeters N, Feng DX, Khounlotham M, Boucher C, Somssich I, Genin S, Marco Y 2003 Physical interaction between RRS1-R, a protein conferring resistance to bacterial wilt, and PopP2, a type III effector targeted to the plant nucleus. Proceedings of the National Academy of Sciences, USA 100, 8024–8029. 10.1073/pnas.1230660100 PMC164706 12788974

[CIT0014] Deslandes L, Olivier J, Theulieres F, Hirsch J, Feng DX, Bittner-Eddy P, Beynon J, Marco Y 2002 Resistance to *Ralstonia solanacearum* in *Arabidopsis thaliana* is conferred by the recessive *RRS1-R* gene, a member of a novel family of resistance genes. Proceedings of the National Academy of Sciences, USA 99, 2404–2409. 10.1073/pnas.032485099 PMC122377 11842188

[CIT0015] Desveaux D, Després C, Joyeux A, Subramaniam R, Brisson N 2000 PBF-2 is a novel single-stranded DNA binding factor implicated in *PR-10a* gene activation in potato. The Plant Cell 12, 1477–1489. 10948264 10.1105/tpc.12.8.1477 PMC149117

[CIT0016] Desveaux D, Maréchal A, Brisson N 2005 Whirly transcription factors: defense gene regulation and beyond. Trends in Plant Science 10, 95–102. 15708347 10.1016/j.tplants.2004.12.008

[CIT0017] Dodds PN, Rathjen JP 2010 Plant immunity: towards an integrated view of plant–pathogen interactions. Nature Reviews Genetics 11, 539–548. 10.1038/nrg2812 20585331

[CIT0018] Feiz L, Williams-Carrier R, Belcher S, Montano M, Barkan A, Stern DB 2014 A protein with an inactive pterin-4a-carbinolamine dehydratase domain is required for Rubisco biogenesis in plants. The Plant Journal 80, 862–869. 25279696 10.1111/tpj.12686

[CIT0019] Fristedt R, Hu C, Wheatley N, et al 2018 RAF2 is a Rubisco assembly factor in *Arabidopsis thaliana*. The Plant Journal 94, 146–156. 29396988 10.1111/tpj.13849

[CIT0020] Fusaro AF, Correa RL, Nakasugi K, Jackson C, Kawchuk L, Vaslin MF, Waterhouse PM 2012 The *Enamovirus* P0 protein is a silencing suppressor which inhibits local and systemic RNA silencing through AGO1 degradation. Virology 426, 178–187. 22361475 10.1016/j.virol.2012.01.026

[CIT0021] García AV, Blanvillain-Baufumé S, Huibers RP, Wiermer M, Li G, Gobbato E, Rietz S, Parker JE 2010 Balanced nuclear and cytoplasmic activities of EDS1 are required for a complete plant innate immune response. PLoS Pathogens 6, e1000970. 20617163 10.1371/journal.ppat.1000970 PMC2895645

[CIT0022] Goodin MM, Dietzgen RG, Schichnes D, Ruzin S, Jackson AO 2002 pGD vectors: versatile tools for the expression of green and red fluorescent protein fusions in agroinfiltrated plant leaves. The Plant Journal 31, 375–383. 12164816 10.1046/j.1365-313x.2002.01360.x

[CIT0023] Gu Y, Dong X 2015 Stromules: signal conduits for plant immunity. Developmental Cell 34, 3–4. 26151902 10.1016/j.devcel.2015.06.018

[CIT0024] Han YH, Xiang HY, Wang Q, Li YY, Wu WQ, Han CG, Li DW, Yu JL 2010 Ring structure amino acids affect the suppressor activity of *Melon aphid-borne yellows virus* P0 protein. Virology 406, 21–27. 20667575 10.1016/j.virol.2010.06.045

[CIT0025] Holsters M, de Waele D, Depicker A, Messens E, van Montagu M, Schell J 1978 Transfection and transformation of *Agrobacterium tumefaciens*. Molecular & General Genetics 163, 181–187. 355847 10.1007/BF00267408

[CIT0026] Holzinger A, Buchner O, Lütz C, Hanson MR 2007 Temperature-sensitive formation of chloroplast protrusions and stromules in mesophyll cells of *Arabidopsis thaliana*. Protoplasma 230, 23–30. 17351732 10.1007/s00709-006-0222-y

[CIT0027] Isemer R, Mulisch M, Schäfer A, Kirchner S, Koop HU, Krupinska K 2012 Recombinant Whirly1 translocates from transplastomic chloroplasts to the nucleus. FEBS Letters 586, 85–88. 22154598 10.1016/j.febslet.2011.11.029

[CIT0028] Jiménez I, López L, Alamillo JM, Valli A, García JA 2006 Identification of a *Plum pox virus* CI-interacting protein from chloroplast that has a negative effect in virus infection. Molecular Plant-Microbe Interactions 19, 350–358. 16570664 10.1094/MPMI-19-0350

[CIT0029] Jin Y, Ma D, Dong J, Li D, Deng C, Jin J, Wang T 2007 The HC-pro protein of *Potato virus Y* interacts with NtMinD of tobacco. Molecular Plant-Microbe Interactions 20, 1505–1511. 17990958 10.1094/MPMI-20-12-1505

[CIT0030] Kong L, Wu J, Lu L, Xu Y, Zhou X 2014 Interaction between *Rice stripe virus* disease-specific protein and host PsbP enhances virus symptoms. Molecular Plant 7, 691–708. 24214893 10.1093/mp/sst158

[CIT0031] Koussevitzky S, Nott A, Mockler TC, Hong F, Sachetto-Martins G, Surpin M, Lim J, Mittler R, Chory J 2007 Signals from chloroplasts converge to regulate nuclear gene expression. Science 316, 715–719. 17395793

[CIT0032] Kozlowska-Makulska A, Guilley H, Szyndel MS, Beuve M, Lemaire O, Herrbach E, Bouzoubaa S 2010 P0 proteins of European beet-infecting poleroviruses display variable RNA silencing suppression activity. The Journal of General Virology 91, 1082–1091. 19955562 10.1099/vir.0.016360-0

[CIT0033] Krause K, Oetke S, Krupinska K 2012 Dual targeting and retrograde translocation: regulators of plant nuclear gene expression can be sequestered by plastids. International Journal of Molecular Sciences 13, 11085–11101. 23109840 10.3390/ijms130911085 PMC3472732

[CIT0034] Kwok EY, Hanson MR 2004 Plastids and stromules interact with the nucleus and cell membrane in vascular plants. Plant Cell Reports 23, 188–195. 15252692 10.1007/s00299-004-0824-9

[CIT0035] Lai Z, Li Y, Wang F, Cheng Y, Fan B, Yu JQ, Chen Z 2011 *Arabidopsis* sigma factor binding proteins are activators of the WRKY33 transcription factor in plant defense. The Plant Cell 23, 3824–3841. 21990940 10.1105/tpc.111.090571 PMC3229152

[CIT0036] Liu Y, Schiff M, Dinesh-Kumar SP 2002a Virus-induced gene silencing in tomato. The Plant Journal 31, 777–786. 12220268 10.1046/j.1365-313x.2002.01394.x

[CIT0037] Liu Y, Schiff M, Marathe R, Dinesh-Kumar SP 2002b Tobacco *Rar1*, *EDS1* and *NPR1/NIM1* like genes are required for *N*-mediated resistance to *Tobacco mosaic virus*. The Plant Journal 30, 415–429. 12028572 10.1046/j.1365-313x.2002.01297.x

[CIT0038] Maleck K, Levine A, Eulgem T, Morgan A, Schmid J, Lawton KA, Dangl JL, Dietrich RA 2000 The transcriptome of *Arabidopsis thaliana* during systemic acquired resistance. Nature Genetics 26, 403–410. 11101835 10.1038/82521

[CIT0039] Mangwende T, Wang ML, Borth W, Hu J, Moore PH, Mirkov TE, Albert HH 2009 The P0 gene of *Sugarcane yellow leaf virus* encodes an RNA silencing suppressor with unique activities. Virology 384, 38–50. 19046592 10.1016/j.virol.2008.10.034

[CIT0040] McGee MD, Rillo R, Anderson AS, Starr DA 2006 UNC-83 is a KASH protein required for nuclear migration and is recruited to the outer nuclear membrane by a physical interaction with the SUN protein UNC-84. Molecular Biology of the Cell 17, 1790–1801. 16481402 10.1091/mbc.E05-09-0894 PMC1415293

[CIT0041] Mou Z, Fan W, Dong X 2003 Inducers of plant systemic acquired resistance regulate NPR1 function through redox changes. Cell 113, 935–944. 12837250 10.1016/s0092-8674(03)00429-x

[CIT0042] Naponelli V, Noiriel A, Ziemak MJ, et al 2008 Phylogenomic and functional analysis of pterin-4a-carbinolamine dehydratase family (COG2154) proteins in plants and microorganisms. Plant Physiology 146, 1515–1527. 18245455 10.1104/pp.107.114090 PMC2287330

[CIT0043] Oh TR, Kim JH, Cho SK, Ryu MY, Yang SW, Kim WT 2017 AtAIRP2 E3 ligase affects ABA and high-salinity responses by stimulating its ATP1/SDIRIP1 substrate turnover. Plant Physiology 174, 2515–2531. 28626006 10.1104/pp.17.00467 PMC5543955

[CIT0044] Pazhouhandeh M, Dieterle M, Marrocco K, et al 2006 F-box-like domain in the polerovirus protein P0 is required for silencing suppressor function. Proceedings of the National Academy of Sciences, USA 103, 1994–1999. 10.1073/pnas.0510784103 PMC1413668 16446454

[CIT0045] Peter KA, Gildow F, Palukaitis P, Gray SM 2009 The C terminus of the polerovirus P5 readthrough domain limits virus infection to the phloem. Journal of Virology 83, 5419–5429. 19297484 10.1128/JVI.02312-08 PMC2681936

[CIT0046] Pfeffer S, Dunoyer P, Heim F, Richards KE, Jonard G, Ziegler-Graff V 2002 P0 of *Beet western yellows virus* is a suppressor of posttranscriptional gene silencing. Journal of Virology 76, 6815–6824. 12050394 10.1128/JVI.76.13.6815-6824.2002 PMC136274

[CIT0047] Pogson BJ, Woo NS, Förster B, Small ID 2008 Plastid signalling to the nucleus and beyond. Trends in Plant Science 13, 602–609. 18838332 10.1016/j.tplants.2008.08.008

[CIT0048] Rhee KH, Stier G, Becker PB, Suck D, Sandaltzopoulos R 1997 The bifunctional protein DCoH modulates interactions of the homeodomain transcription factor HNF1 with nucleic acids. Journal of Molecular Biology 265, 20–29. 8995521 10.1006/jmbi.1996.0708

[CIT0049] Scagliusi SM, Lockhart BE 2000 Transmission, characterization, and serology of a luteovirus associated with yellow leaf syndrome of sugarcane. Phytopathology 90, 120–124. 18944599 10.1094/PHYTO.2000.90.2.120

[CIT0050] Schattat M, Barton K, Baudisch B, Klösgen RB, Mathur J 2011 Plastid stromule branching coincides with contiguous endoplasmic reticulum dynamics. Plant Physiology 155, 1667–1677. 21273446 10.1104/pp.110.170480 PMC3091094

[CIT0051] Shen QH, Saijo Y, Mauch S, Biskup C, Bieri S, Keller B, Seki H, Ulker B, Somssich IE, Schulze-Lefert P 2007 Nuclear activity of MLA immune receptors links isolate-specific and basal disease-resistance responses. Science 315, 1098–1103. 17185563 10.1126/science.1136372

[CIT0052] Shi Y, Chen J, Hong X, Chen J, Adams MJ 2007 A potyvirus P1 protein interacts with the Rieske Fe/S protein of its host. Molecular Plant Pathology 8, 785–790. 20507538 10.1111/j.1364-3703.2007.00426.x

[CIT0053] Stevens M, Freeman B, Liu HY, Herrbach E, Lemaire O 2005 Beet poleroviruses: close friends or distant relatives?Molecular Plant Pathology 6, 1–9. 20565633 10.1111/j.1364-3703.2004.00258.x

[CIT0054] Suck D, Ficner R 1996 Structure and function of PCD/DCoH, an enzyme with regulatory properties. FEBS Letters 389, 35–39. 8682201 10.1016/0014-5793(96)00573-x

[CIT0055] Sun X, Feng P, Xu X, Guo H, Ma J, Chi W, Lin R, Lu C, Zhang L 2011 A chloroplast envelope-bound PHD transcription factor mediates chloroplast signals to the nucleus. Nature Communications 2, 477. 10.1038/ncomms1486 21934661

[CIT0056] Taliansky M, Mayo MA, Barker H 2003 *Potato leafroll virus*: a classic pathogen shows some new tricks. Molecular Plant Pathology 4, 81–89. 20569366 10.1046/j.1364-3703.2003.00153.x

[CIT0057] Tao Y, Xie Z, Chen W, Glazebrook J, Chang HS, Han B, Zhu T, Zou G, Katagiri F 2003 Quantitative nature of *Arabidopsis* responses during compatible and incompatible interactions with the bacterial pathogen *Pseudomonas syringae*. The Plant Cell 15, 317–330. 12566575 10.1105/tpc.007591 PMC141204

[CIT0058] Valkai I 2004 28D, a new component of the phytochrome B signal transduction, in *Arabidopsis thaliana*. Acta Biologica Szegediensis 48, 87.

[CIT0059] Wang D, Amornsiripanitch N, Dong X 2006 A genomic approach to identify regulatory nodes in the transcriptional network of systemic acquired resistance in plants. PloS Pathogens 2, e123. 17096590 10.1371/journal.ppat.0020123 PMC1635530

[CIT0060] Wang KD, Empleo R, Nguyen TT, Moffett P, Sacco MA 2015 Elicitation of hypersensitive responses in *Nicotiana glutinosa* by the suppressor of RNA silencing protein P0 from poleroviruses. Molecular Plant Pathology 16, 435–448. 25187258 10.1111/mpp.12201 PMC6638411

[CIT0061] Wen W, Meinkoth JL, Tsien RY, Taylor SS 1995 Identification of a signal for rapid export of proteins from the nucleus. Cell 82, 463–473. 7634336 10.1016/0092-8674(95)90435-2

[CIT0062] Win J, Kamoun S, Jones AM 2011 Purification of effector-target protein complexes via transient expression in *Nicotiana benthamiana*. Methods in Molecular Biology 712, 181–194. 21359809 10.1007/978-1-61737-998-7_15

[CIT0063] Xiang HY, Dong SW, Shang QX, Zhou CJ, Li DW, Yu JL, Han CG 2011 Molecular characterization of two genotypes of a new polerovirus infecting brassicas in China. Archives of Virology 156, 2251–2255. 21874520 10.1007/s00705-011-1091-z

[CIT0064] Xiang HY, Han CG 2011 Molecular characterization of novel poleroviruses and poleroviral P0 protein functional analysis. Acta Phytopathologica Sinica 41 (ZK), 177–178 (in Chinese).

[CIT0065] Yang H, Shi Y, Liu J, Guo L, Zhang X, Yang S 2010 A mutant CHS3 protein with TIR-NB-LRR-LIM domains modulates growth, cell death and freezing tolerance in a temperature-dependent manner in *Arabidopsis*. The Plant Journal 63, 283–296. 20444230 10.1111/j.1365-313X.2010.04241.x

[CIT0066] Yao M, Zhang T, Tian Z, Wang Y, Tao X 2011 Construction of *Agrobacterium*-mediated *Cucumber mosaic virus* infectious cDNA clones and 2b deletion viral vector. Scientia Agricultura Sinica 44, 4886–4890.

[CIT0067] Zhang H, Cui F, Wu Y, Lou L, Liu L, Tian M, Ning Y, Shu K, Tang S, Xie Q 2015a The RING finger ubiquitin E3 ligase SDIR1 targets SDIR1-INTERACTING PROTEIN1 for degradation to modulate the salt stress response and ABA signaling in *Arabidopsis*. The Plant Cell 27, 214–227. 25616872 10.1105/tpc.114.134163 PMC4330582

[CIT0068] Zhang XY, Dong SW, Xiang HY, Chen XR, Li DW, Yu JL, Han CG 2015b Development of three full-length infectious cDNA clones of distinct brassica yellows virus genotypes for *Agrobacterium*-mediated inoculation. Virus Research 197, 13–16. 25499296 10.1016/j.virusres.2014.12.005

[CIT0069] Zhang XY, Xiang HY, Zhou CJ, Li DW, Yu JL, Han CG 2014 Complete genome sequence analysis identifies a new genotype of brassica yellows virus that infects cabbage and radish in China. Archives of Virology 159, 2177–2180. 24599564 10.1007/s00705-014-2027-1

[CIT0070] Zhang Y, Fan W, Kinkema M, Li X, Dong X 1999 Interaction of NPR1 with basic leucine zipper protein transcription factors that bind sequences required for salicylic acid induction of the *PR-1* gene. Proceedings of the National Academy of Sciences, USA 96, 6523–6528. 10.1073/pnas.96.11.6523 PMC26915 10339621

[CIT0071] Zhao J, Zhang X, Hong Y, Liu Y 2016 Chloroplast in plant–virus interaction. Frontiers in Microbiology 7, 1565. 27757106 10.3389/fmicb.2016.01565 PMC5047884

[CIT0072] Zhuo T, Li YY, Xiang HY, Wu ZY, Wang XB, Wang Y, Zhang YL, Li DW, Yu JL, Han CG 2014 Amino acid sequence motifs essential for P0-mediated suppression of RNA silencing in an isolate of *Potato leafroll virus* from Inner Mongolia. Molecular Plant-Microbe Interactions 27, 515–527. 24450775 10.1094/MPMI-08-13-0231-R

[CIT0073] Zybailov B, Rutschow H, Friso G, Rudella A, Emanuelsson O, Sun Q, van Wijk KJ 2008 Sorting signals, N-terminal modifications and abundance of the chloroplast proteome. PloS ONE 3, e1994. 18431481 10.1371/journal.pone.0001994 PMC2291561

