# Supplementary material for: Brassica yellows virus P0 protein impairs the antiviral activity of NbRAF2 in Nicotiana benthamiana
Source: J Exp Bot. 2018 Apr 5;69(12):3127–39. doi: 10.1093/jxb/ery131 (PMC5972614; doi:10.1093/jxb/ery131)
Supplement: Supplementary Table S1 [file ery131_suppl_supplementary_table_s1.pdf]

Table S1. List of primers used in this study

Experiment	Name	Primers Sequence (5'-3')	Note
Co-IP/ Nucleocytoplasmic fractionation	P38-F	CCCAAGCTTATGGAAAATGATCCTAGAG	For pGD
	P38-R	CGCGGATCCCTAAATTCTGAGTGCTTG	
	BrAP0-XhoIF	TCTCGAGATGCAATTTGTAGC	pGD-3Flag
	BrAP0-ApaIR	CGGGCCCACAAACATTTCGG	
	RbcL-XhoIF	TATCTCGAGATGAGTTGTAGGGAGGGATT	
	RbcL-ApaIR	TATGGGCCCCTTATCCAAAACGTCCACTG	
	PLPO-F1	TCGAGATGATTGTATTGACCCAG	
	PLPO-R1	GGCCCTTCTTGTAATTCCTTTT	
	PLPO-F2	GATGATTGTATTGACCCAG	
	PLPO-R2	CTTCTTGTAATTCCTTTT	
	NbRAF2-XhoINdeIF	TATCATATGCTCGAGATGGCTACTGCAACTCATT	pGDGm
	NbRAF2-ApaIR	TATGGGCCCTGCCCAAACTCTTTTTCTTG GG	
	AtRAF2-NdeIXhoIF	TATCATATGCTCGAGATGGCCGCCACGTCATC	
	AtRAF2-ApaIR	TATGGGCCCCGCCCAAGCTCTTTTCC	
	NbSKP1-XhoIF	CCGCTCTGAGATGAAGATGATCGTGC	
	NbSKP1-ApaIR	ACGGGCCCCTCGAAGGCCCAGGC	
Confocol/ Nucleocytoplasmic fractionation/ Overexpression	NbRAF2-NdeIXhoIF	TATCATATGCTCGAGATGGCTACTGCAACTCATT	pGD-3G-mCherry
	NbRAF2-ApaIR	TATGGGCCCTGCCCAAACTCTTTTTCTTGGG	
	NLS-NbRAF2-ΔTP-XhoIF	TATCTCGAGATGCCAAAAAAGAAGAGAAAGGTAGGAGACCTATTGGGCGAT	
	NbRAF2-ApaIR	TATGGGCCCTGCCCAAACTCTTTTTCTTGGG	
	AtRAF2-NdeIXhoIF	TATCATATGCTCGAGATGGCCGCCACGTCATC	
	AtRAF2-ApaIR	TATGGGCCCCGCCCAAGCTCTTTTCC	
	AtRAF2-ΔTP-XhoIF	TTCTCGAGCATATGTCTAATCTGGCGCAGGATTTT	
	AtRAF2-ApaIR	TATGGGCCCCGCCCAAGCT CTTTTCC	
	BrAP0-SalIF	GGGTCGACATGCAATTTGTAGCTCACG	pSuper1300-GFP
	BrAP0-KpnIR	ACGCGGTACCTACAAACATTTCGGTGTAG	

Experiment	Name	Primers Sequence (5'-3')	Note
BiFC	NbRAF2-SpeIF	GGACTAGTATGGCTACTGCAACTCATT	pSPYNE35S/ pSPYCE-35S
	NbRAF2-SalIR	TATGTCGACTGCCCAAACTCTTTTTCT	
	NbRAF2-ΔCter-SalIR	TATGTCGACTCCGATGGAAGCAGTCCA	
	AtRAF2-SpeIF	GGACTAGTATGGCCGCCACGTCATC	
	AtRAF2-SalIR	TATGTCGACCGCCCAAGCTCTTTTCC	
	AtRAF2-ΔCter-SalIR	TATGTCGACTCCGATAGAAGAGGTAAA	
TRV-VIGS	NbRAF2-160XhoI	TATCTCGAGCGAGAGATCCATTCCCAGC	pTRV2
	NbRAF2-540BamHI	TATGGATCCGGAAGCAGTCCATAGCTC	
	GFP-XhoI	CCGCTCGAGTCAAGAGTGCCATGCCCGAA	
	GFP-BamHI	CGCGGATCCCATCCATGCCATGTGTAATCCCA	
	NbRAF2-1F	TCCCTCCACCAATTTCAGTC	For semi-quantitative RT-PCR
	NbRAF2-1R	AATCGCCCAATAGGTCTCCC	
	EF1AF	AGCTTTACCTCCCAAGTCATC	
	EF1AR	AGAACGCCTGTCAATCTTGG	
	PoconF	GAYTGYTCYGGTTTTGACTGG	For BrYV-A RNA detection
	PoconR	CGTCTACCTATTTSGGRTTN	
Y2H	NbRAF2-NdeIF	TATCATATGGCTACTGCAACTCATT	pGDBKT7/ pGDADT7
	NbRAF2-BamHIR	TATGGATCCTCATGCCCAAACTCTTTTTCTTG	
	AtRAF2-NdeIF	TATCATATGGCCGCCACGTCATC	
	AtRAF2-XhoIR	CCGCTCGAGTCACGCCCAAGCTCTTTTCC	
	NbRAF2-ΔTP-XhoINdeIF	TTCTCGAGCATATGGCTGAAATAGAGAGTAAATTC	pGDADT7
	NbRAF2-ΔCter-BamHIR	TATGGATTCTCATCCGATGGAAGCAGTCCA	
	AtRAF2-ΔCter-BamHIR	TATGGATTCTCATCCGATAGAAGAGGTAAA	
	NbRbcL-EcoRIF	TATGAATTCATGAGTTGTAGGGAGGGATT	pGDBKT7
	NbRbcL-SalIR	TATGTCGACTTACTTATCCAAAACGTCCACTG	
	BrAP0-NdeIF	TTCCATATGATGCAATTTGTAGCTCAC	
	BrAP0-BamHIR	CGGGATCCTACAAACATTTCGGTGT	
	P0^{MA}-NdeIF	CCATATGACAAAAGATACGAGCGGGTG	

Experiment	Name	Primers Sequence (5'-3')	Note
	P0^{MA}-SalIR	GCGTCGACTAATGGTGGTGGTGGTGATG	
	P0^{Sc}-BamHF	CGGGATCCATGCTTTTCAACGAATTC	
	P0^{Sc}-SalIR	ACGCGTCGACTATATCATGAGAATAGGTG	
MbYTH	NbRAF2-BT3F	ATTAACAAGGCCATTACGGCCGCTACTGCAACTCATT	pBT3-STE
	NbRAF2-BT3R	AACTGATTGGCCGAGGCGGCCCCTGCCCAAACTCTTTTTC	
	BrAP0-PR3F	CGCAGAGTGGCCATTACGGCCATGCAATTTGTAGCTCACG	pPR3-N
	BrAP0-PR3R	CTCGAGAGGCCGAGGCGGCCTCATACAAACATTTCGGTGTAG	
