# Supplementary material for: Brassica yellows virus P0 protein impairs the antiviral activity of NbRAF2 in Nicotiana benthamiana
Source: J Exp Bot. 2018 Apr 5;69(12):3127–39. doi: 10.1093/jxb/ery131 (PMC5972614; doi:10.1093/jxb/ery131)
Supplement: Supplementary Figures S1-S14 [file ery131_suppl_supplementary_figures_s1_s14.pdf]

```
NbRAF2   ..MATATHFCFPPPISVSAQK.....FQSAKFFTPSLQTT     33
AtRAF2   MAATSSSPPCNISASSLLLRQPSRSILKVFGLLPPVSRNN     40
ZmRAF2   ....MALRLSLAPPAAAPAASTCR..CSSASFIRPGVAVG     34
Consensus                                  p

NbRAF2   QSRTCTRILCN..GGDLLGDFGARDPFPAEIESKFGEKVL     71
AtRAF2   RKLGRLTVTRSNLAQDFLGDFGARDPYPEEIASQFGDKVL     80
ZmRAF2   SRRGRARGCVVA.MADLLGDLGARDPFPEEIESQFGEKVL     73
Consensus               d lgd gardp p ei s fg kvl

NbRAF2   GNTDTEHKILIPAASALSLAQQDYTVISPGQTPLSEYEAR    111
AtRAF2   GCQSTEHKILIPNASVLSLSQLQCSPVSSSQPPLSGDDAR    120
ZmRAF2   GNVDTLHQILIPTLSALSLASL...PLQPDAEPLSLDDAR    110
Consensusg   t h ilip  s lsl             pls   ar

NbRAF2   QLLFKVVGWRLAH......EDGVLKLQCTWKLRDFECGVE    145
AtRAF2   TLLHKVLGWSIVDN.....EAGGLKIRCMWKVRDFGCGVE    155
ZmRAF2   RLLFKVVGWRLVLSDGDGDEQRPARLQCVWKVRDETCGQE    150
Consensus ll kv gw          e       c wk rd  cg e

NbRAF2   LINRIGKVVEGTEHVPTLLHLEQSNQVRAELWTASIGG..    183
AtRAF2   LINRIHKVAEASGHYPS.LHLESPTQVRAELFTSSIGG..    192
ZmRAF2   LIARINAALHGAGHAPATLAFEAPNQVTAELSTPSAAGDS    190
Consensusli ri        h p  l  e   qv ael t s  g

NbRAF2   LSINDFIVAAKIDQIKTSDLVPRKRVWA                211
AtRAF2   LSMNDFIMAAKIDDIKTSDLSPRKRAWA                220
ZmRAF2   LTVNDYIVAARIDKVKTLDLIPKKRAWA                218
Consensusl  nd i aa id  kt dl p kr wa
```

Supplementary Figure S1. Multiple sequence alignment of representative RAF2 proteins.

The RAF2 amino acid sequence from *Nicotiana benthamiana* (Nb; MG560271) was aligned with its homolog from *Arabidopsis thaliana* (At; AED96036.1) and *Zea mays* (Zm; NP_001144391).

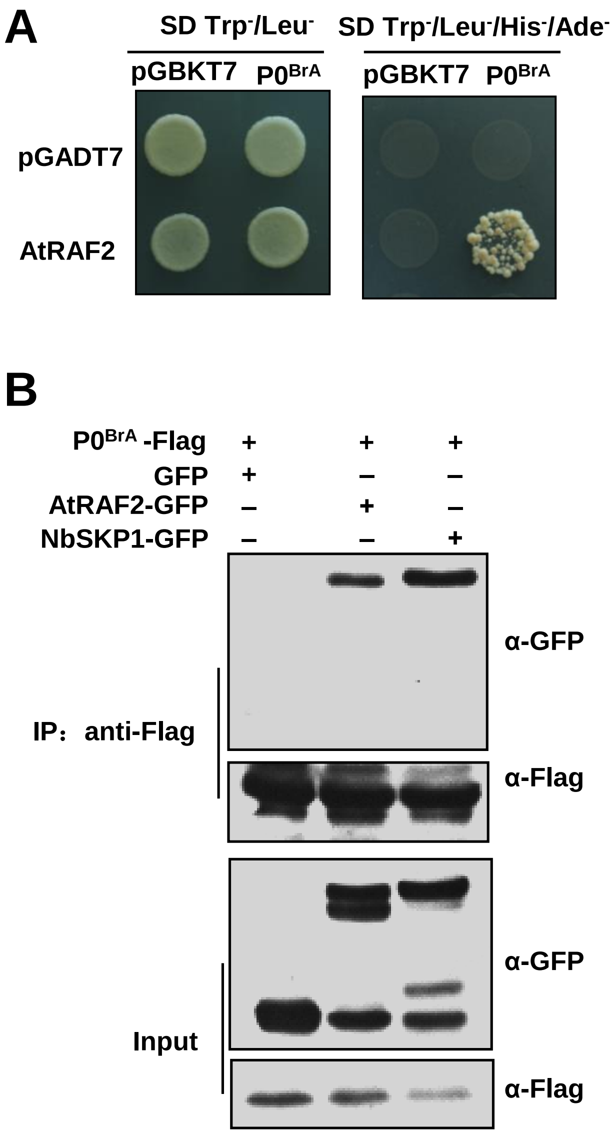

Supplementary Figure S2. AtRAF2 interacts with P0^{BrA}.

(A) Analysis of interactions between P0^{BrA} and AtRAF2 in the yeast two-hybrid system. P0^{BrA} was cloned into bait vector pGBKT7 and transformed into yeast Y187. AtRAF2 was cloned into prey vector pGADT7 and transformed into yeast AH109. Yeast strains were grown on SD/-Leu/-Trp and SD/-Ade/-His/-Leu/-Trp, and maintained at 28℃ for 5–7 d. (B) Co-immunoprecipitation analyses of P0^{BrA} and RAF2 proteins in *N. benthamiana* leaves. P0^{BrA}-Flag was co-expressed with GFP, AtRAF2 and NbSKP1 through agroinfiltration. Protein complexes were immunoprecipitated using anti-Flag beads. Immunoprecipitates were assessed by western blotting using anti-GFP and anti-Flag antibodies.

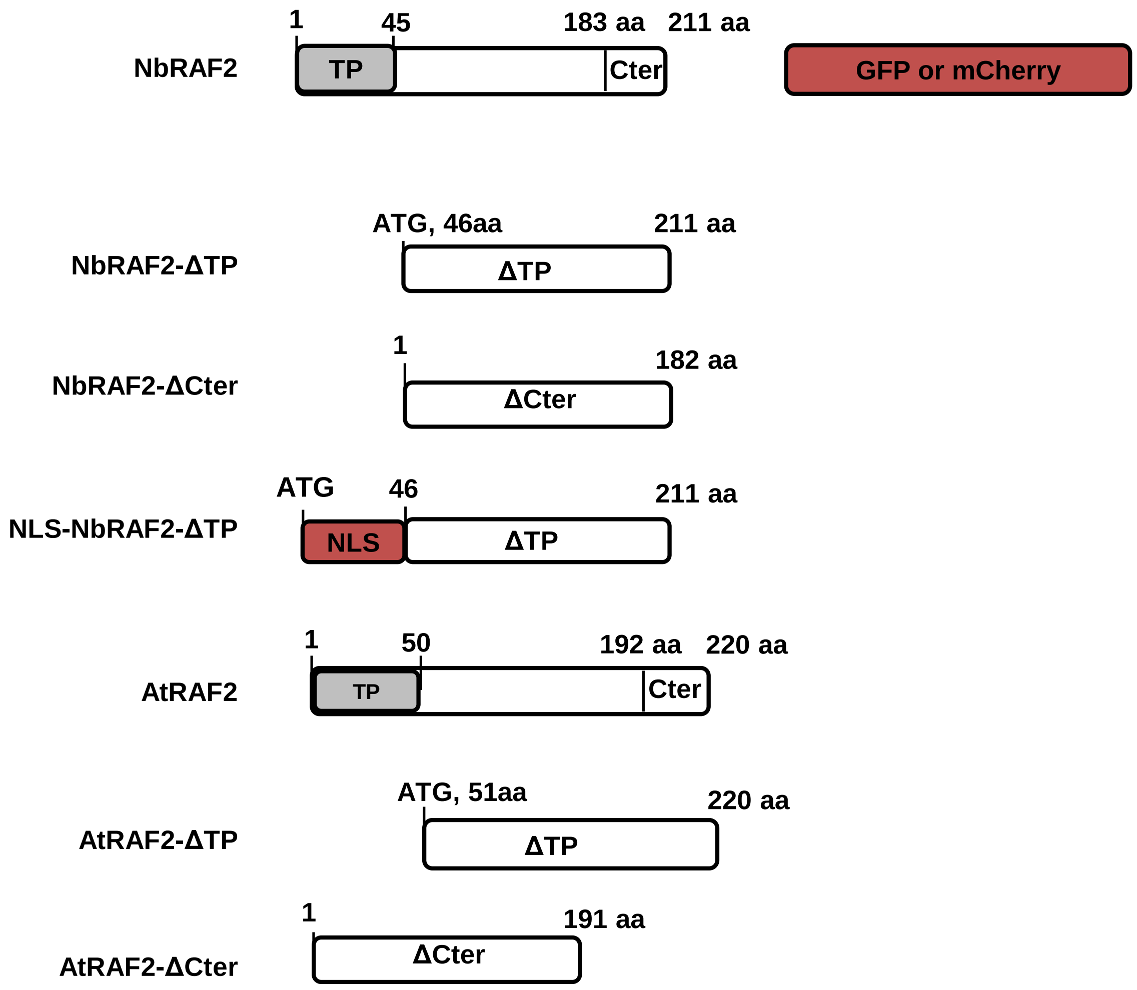

Supplementary Figure S3. NbRAF2, AtRAF2 and their mutant constructs used for confocal microscopy or Y2H.

Constructs containing NbRAF2, NbRAF2 mutants, AtRAF2 and AtRAF2 mutants used for confocal microscopy or Y2H. Both GFP and mCherry fusions were built. TP, transit peptide; Cter, C-terminal residues.

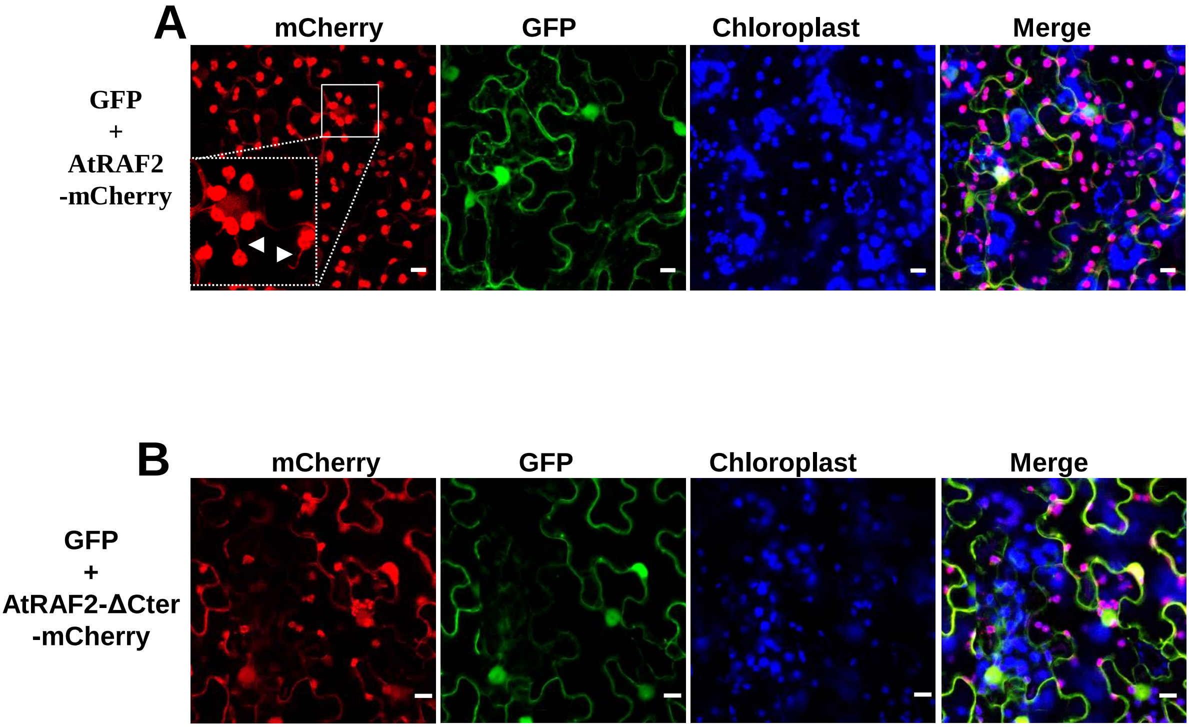

Supplementary Figure S4. The subcellular localization of AtRAF2 and AtRAF2-ΔCter.

(A) AtRAF2 is localized to the soluble, stromal fraction of chloroplasts. AtRAF2-mCherry was transiently co-expressed with GFP in leaves through agroinfiltration and imaged at 2 dpi. Z stacks of cells showing a representative distribution of AtRAF2-mCherry were imaged. White arrows indicate that AtRAF2 localized to the stromules of chloroplasts. Blue represents the autofluorescence of chloroplasts. GFP was used as a nucleocytoplasmic marker. The scale bars represent 10 μm. (B) AtRAF2-ΔCter is located to chloroplasts, nuclei and cell periphery. Blue represents the autofluorescence of chloroplasts. GFP was used as a nucleocytoplasmic marker. The scale bars represent 10 μm.

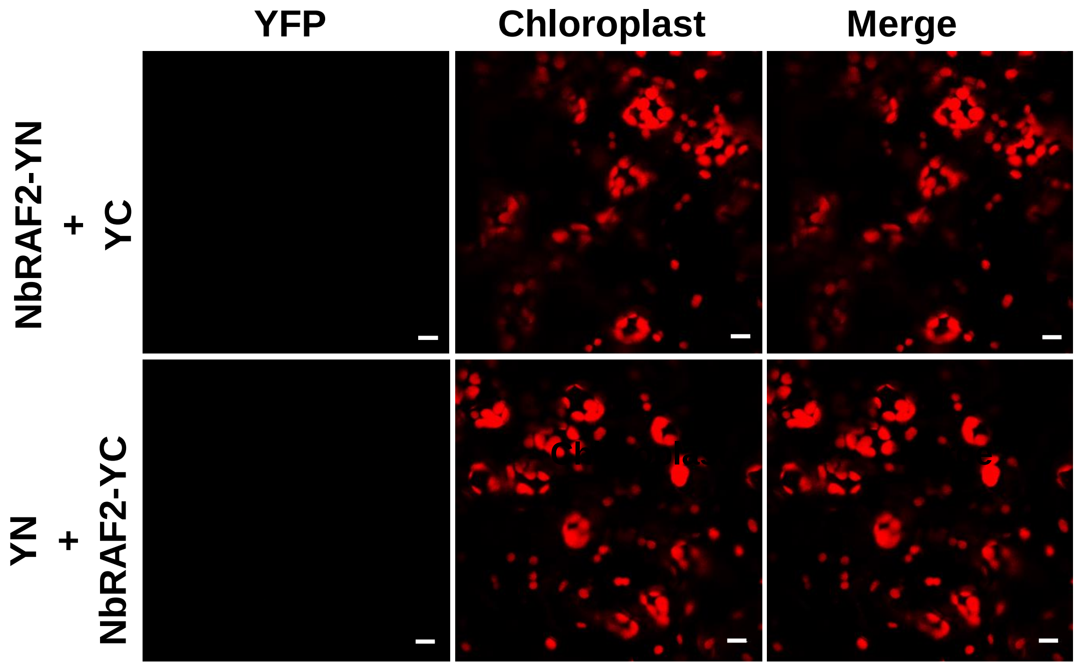

Supplementary Figure S5. Controls for BiFC assays for NbRAF2 self-interaction.

Control samples were expressed in *N. benthamiana* leaves through agroinfiltration. The scale bars represent 10 μm.

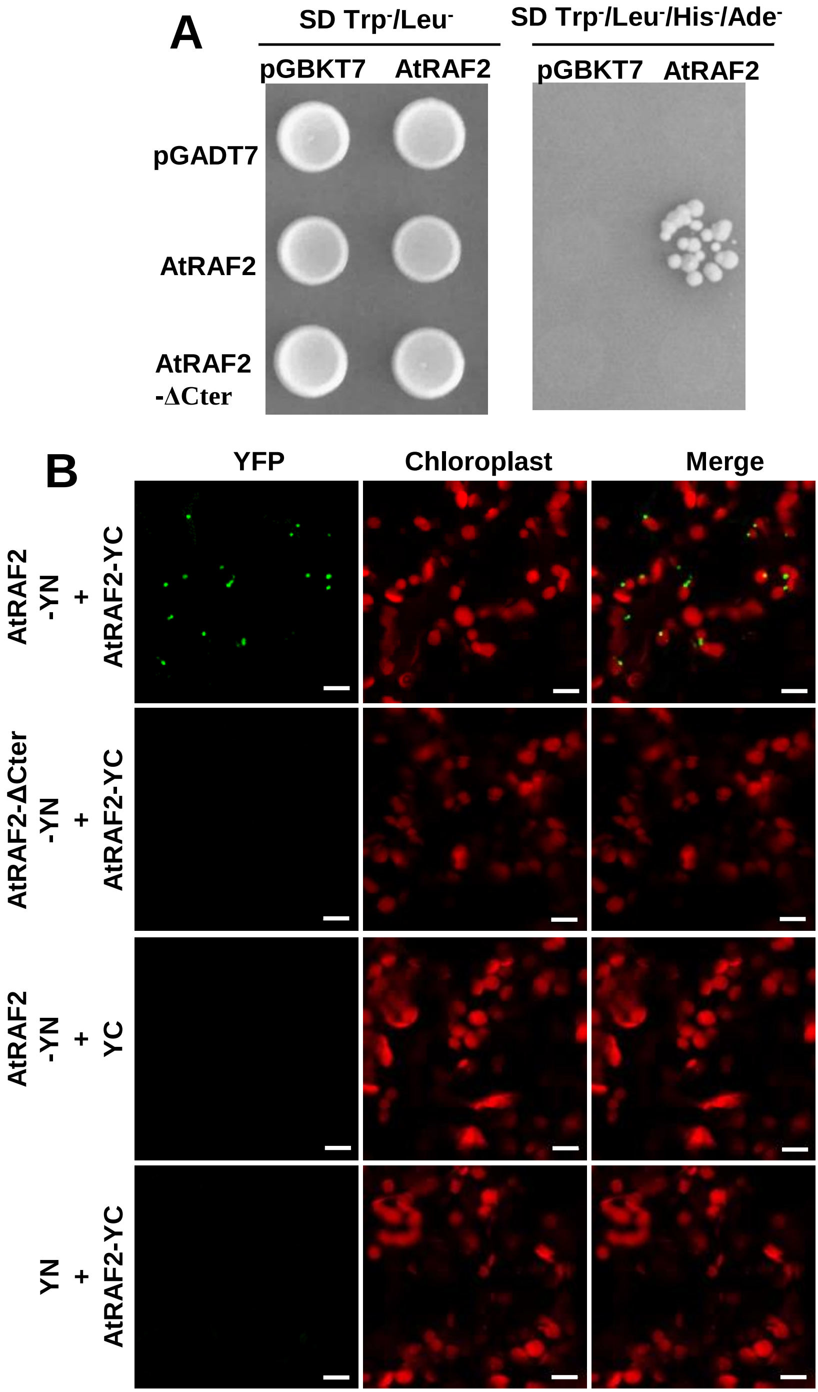

Supplementary Figure S6. Self-interaction of AtRAF2.

(A) Yeast two-hybrid identifying the AtRAF2 self-interaction. AD constructs containing AtRAF2 and its variants were transformed with BD-AtRAF2. Yeast cells transformed separately with BD and AD vectors showed no growth on selective plates. Yeast strains were grown on SD/-Leu/-Trp and SD/-Ade/-His/-Leu/-Trp, and maintained at 28℃ for 5–7 d. (B) BiFC assays were performed on *N. benthamiana* leaves through agroinfiltration. Red fluorescence represents the autofluorescence of chloroplasts. Green fluorescence represents the reconstitution of YFP as an indicator of protein–protein interactions. Images show AtRAF2–AtRAF2 interactions. C-terminal residues are those required for the RAF2 self-interaction.

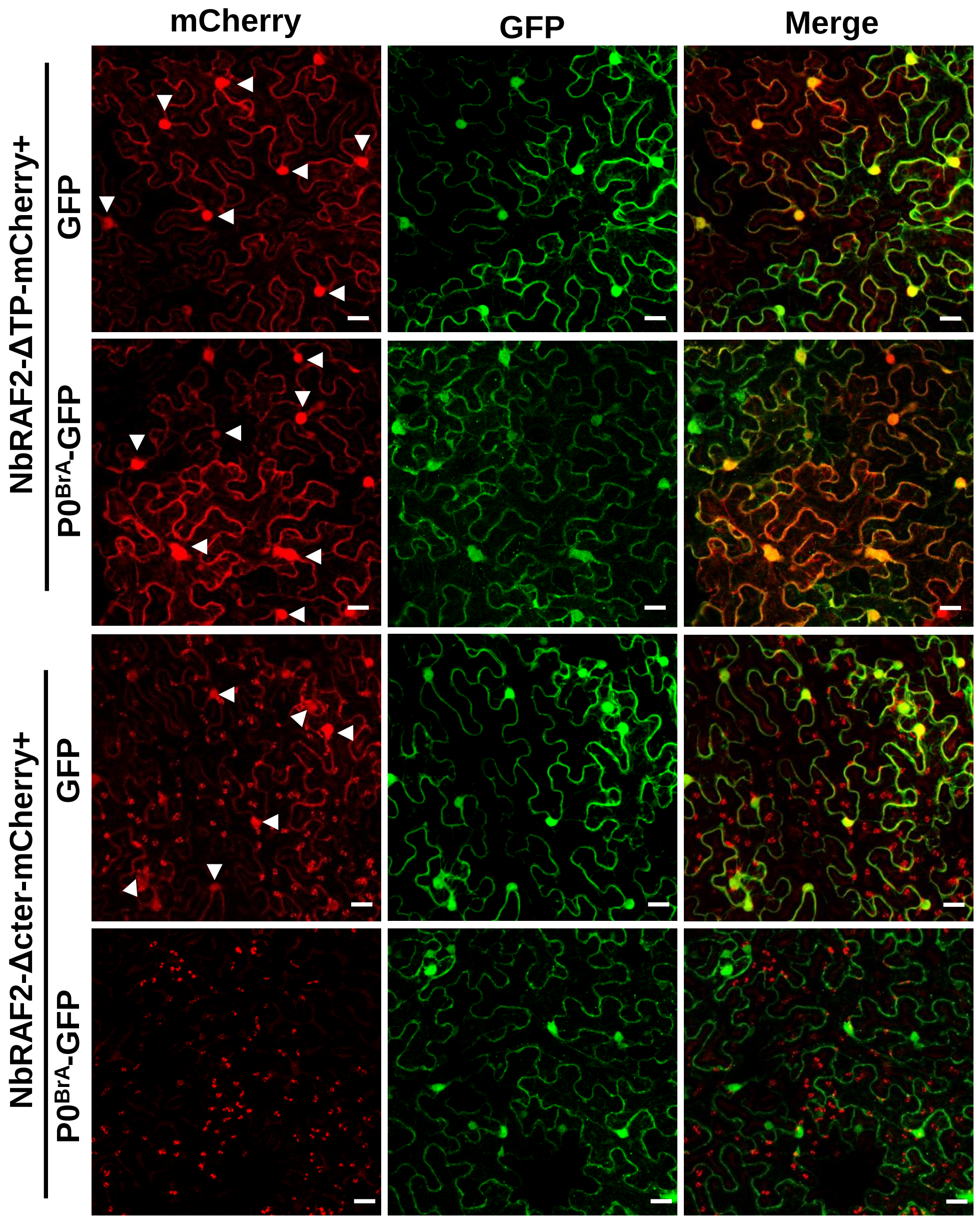

Supplementary Figure S7. P0^{BrA}–GFP decreases the nuclear pool of NbRAF2–ΔCter but not NbRAF2–ΔTP. The localizations of NbRAF2–ΔTP and NbRAF2–ΔCter when coexpressed with GFP or P0^{BrA}–GFP. Images were taken at 2 dpi. The scale bars represent 20 μm. Z stacks of cells were imaged.

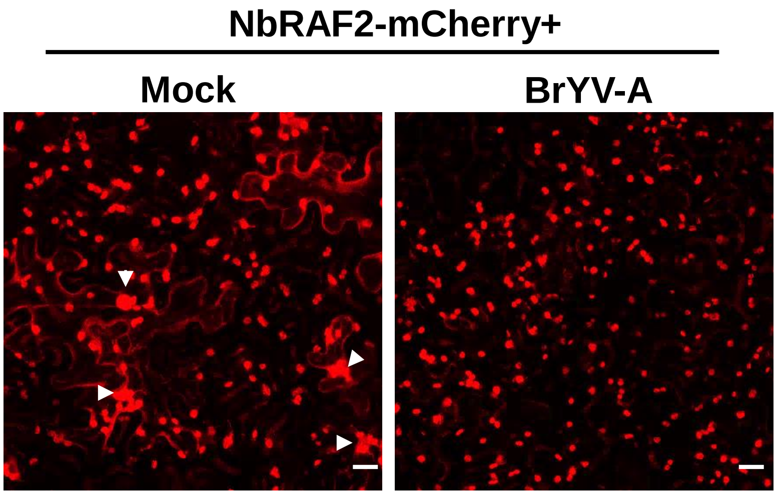

Supplementary Figure S8. The localization of NbRAF2-mCherry when coexpressed with BrYV-A. BrYV-A decreases the nuclear pool of NbRAF2. Images were taken at 2 dpi. Z stacks of cells were imaged. The scale bars represent 20 μm.

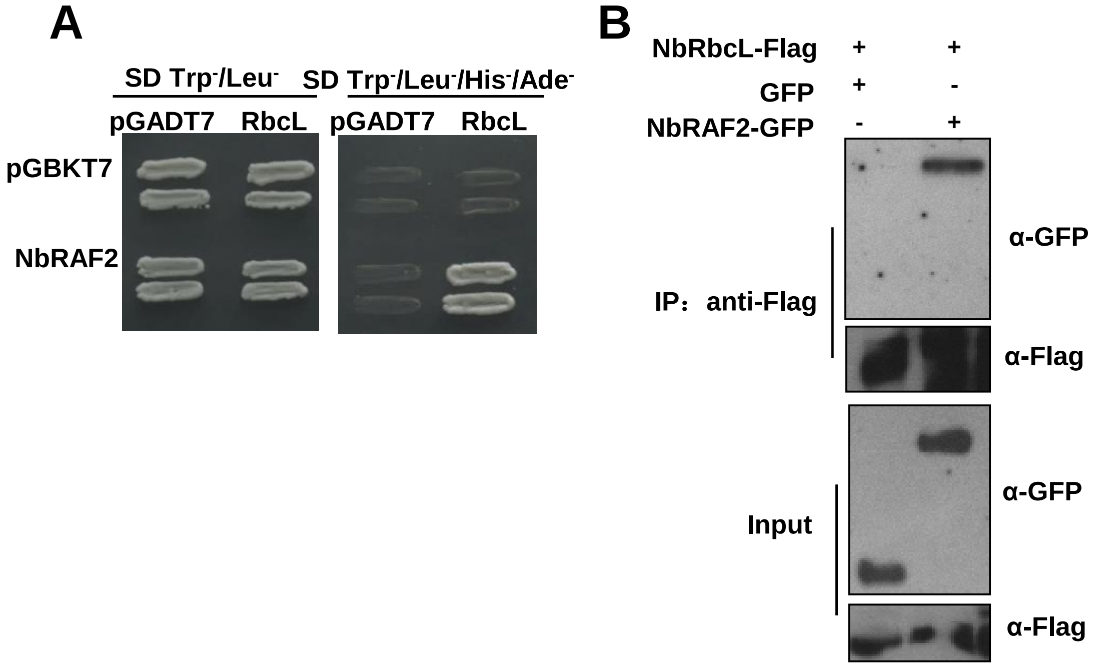

Supplementary Figure S9. NbRAF2 interacts with NbRbcL.

(A) Analysis of interactions between NbRAF2 and NbRbcL in the yeast two-hybrid system. AD constructs containing NbRAF2 were transformed with BD-NbRbcL. Yeast cells transformed separately with BD and AD vectors showed no growth on selective plates. Yeast strains were grown on SD/-Leu/-Trp and SD/-Ade/-His/-Leu/-Trp, and maintained at 28℃ for 5–7 d. (B) Co-immunoprecipitation analyses of NbRAF2 and NbRbcL proteins in *N. benthamiana* leaves. Protein complexes were immunoprecipitated using anti-Flag beads. Immunoprecipitates were assessed by western blotting using anti-GFP or anti-Flag antibodies.

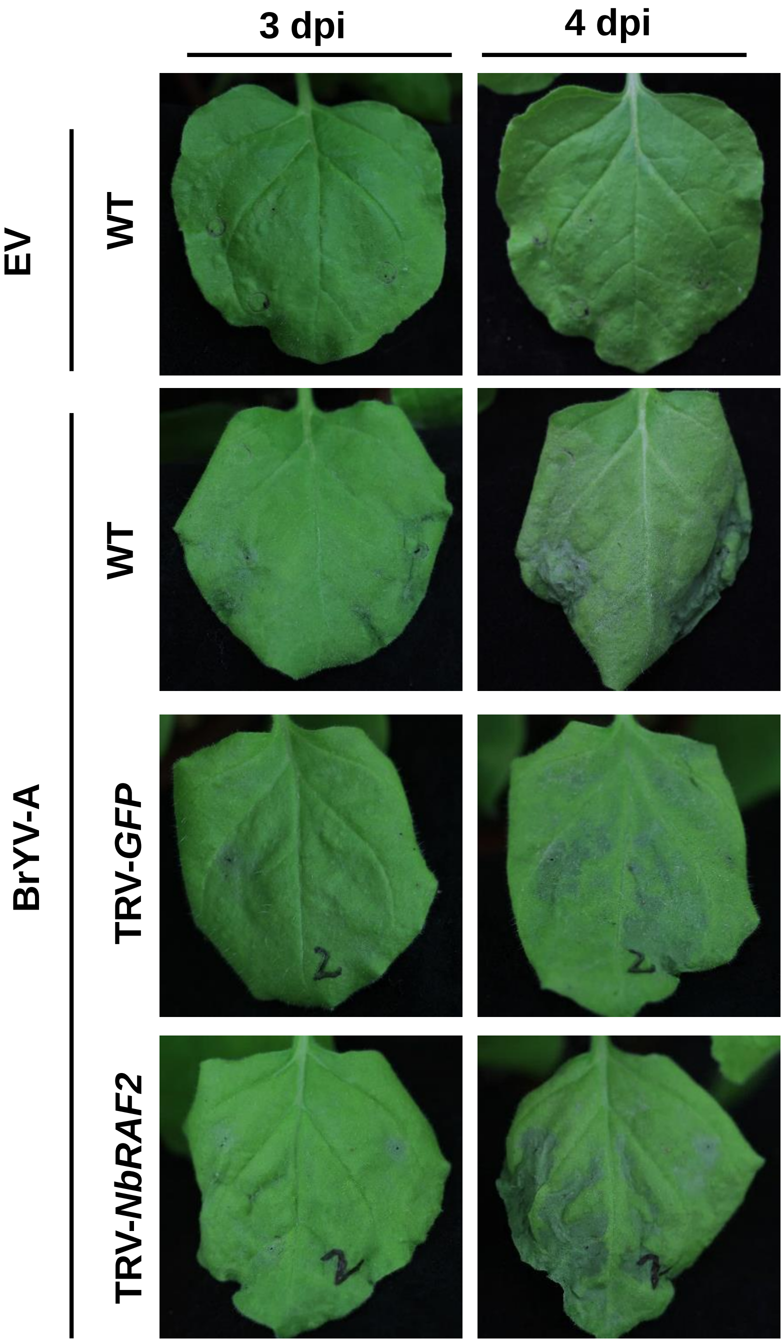

Supplementary Figure S10. Wild-type**,** *NbRAF2*-silenced and non-silenced N. benthamiana were inoculated with mock or BrYV-A. Photos were taken at 3 dpi and 4dpi.

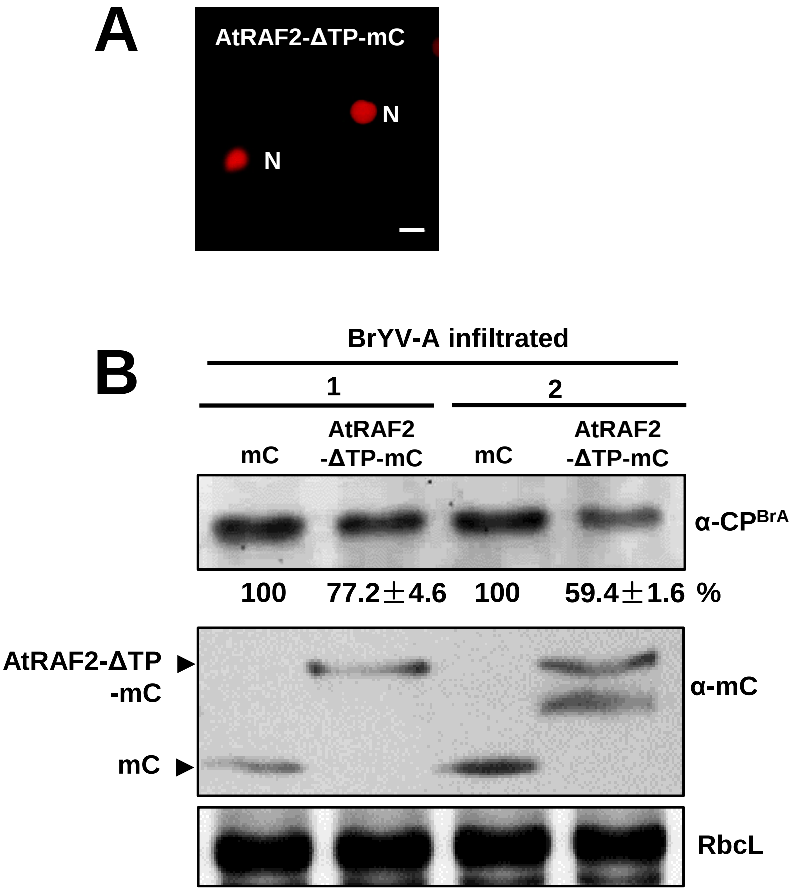

Supplementary Figure S11. Overexpression of nuclear AtRAF2 enhances resistence to BrYV-A. BrYV-A was agroinfiltrated in leaves separately overexpressing AtRAF2–ΔTP–mC and mC through agroinfiltration at 1 dpi. Then BrYV-A CP was detected at 2 d after BrYV-A inoculated. CP was detected with BrYV-A CP polyclonal antiserum. RbcL is the Rubisco large subunit. 1 and 2 represent two plants. ImageJ software was used to quantify the bands.

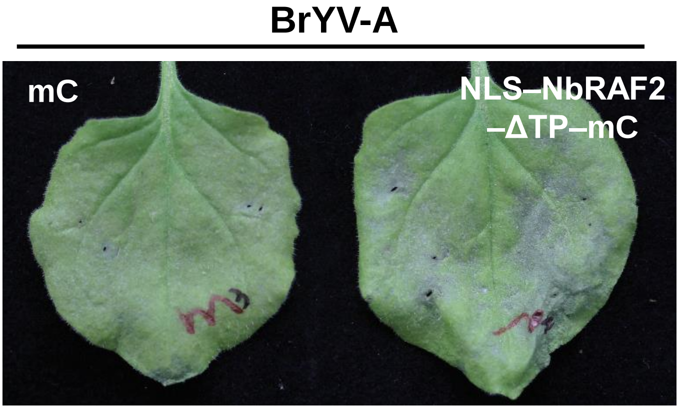

Supplementary Figure S12. overexpression of nuclear NbRAF2 (NLS–NbRAF2–ΔTP–mC) enhances necrosis when inoculated with BrYV-A. Photographs were taken at 4 dpi. mCherry (mC) was used as negative control.

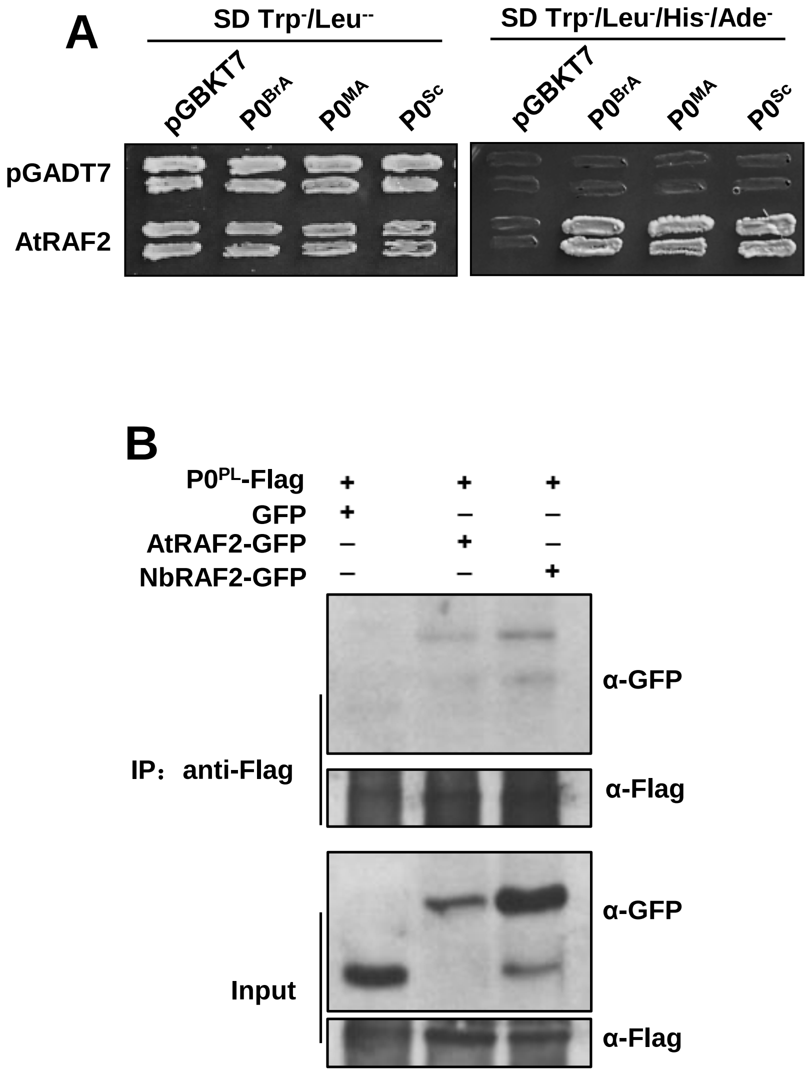

Supplementary Figure S13. Polerovirus P0 proteins interact with AtRAF2 and NbRAF2.

(A) Analysis of interactions between polerovirus P0 and AtRAF2 in the yeast two-hybrid system. Different combinations were transformed. Yeast strains were grown on SD/-Leu/-Trp and SD/-Ade/-His/-Leu/-Trp, and maintained at 28℃ for 5–7 d. (B) Co-immunoprecipitation analyses for interactions of $P0^{PL}$ with NbRAF2 and AtRAF2 in *N. benthamiana* leaves. $P0^{PL}$-Flag was co-expressed with GFP, NbRAF2-GFP and AtRAF2-GFP through agroinfiltration. Protein complexes were immunoprecipitated using anti-Flag beads. Immunoprecipitates were assessed with western blotting using anti-GFP and anti-Flag antibodies

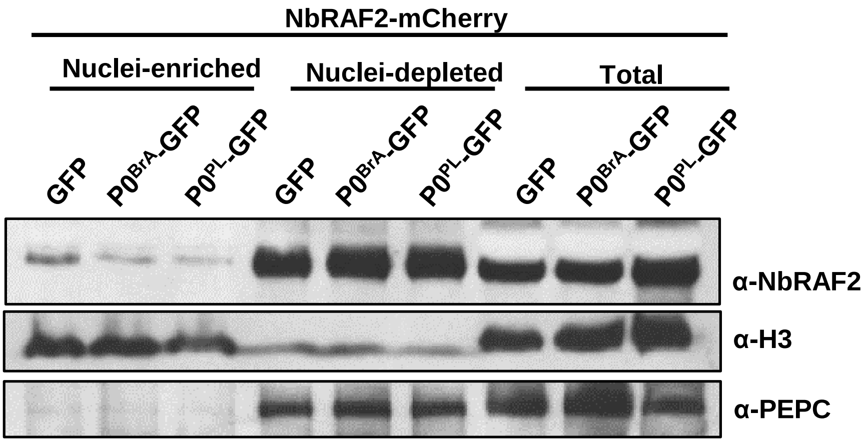

Supplementary Figure S14. PLRV P0 decreases the nuclear enrichment of NbRAF2.

Western blot of NbRAF2 in nuclei-enriched, nuclei-depleted fractions and total protein in *N. benthamiana.* Protein samples were prepared from plant leaves at 2 dpi. All of the fractions were subjected to immunoblot analyses. PEPC and histone H3 signals were used as cytosolic and nuclear markers, respectively.
